# Traditional Chinese medicine remodels the immune microenvironment of colorectal cancer: mechanisms and therapeutic perspectives

**DOI:** 10.3389/fimmu.2026.1932903

**Published:** 2026-09-03

**Authors:** Zeanbang Shi, Jie Mei, Qinchang Zhang, Liu Li, Haibo Cheng

**Affiliations:** 1 The First Clinical Medical College, Nanjing University of Chinese Medicine, Nanjing, China; 2 Jiangsu Collaborative Innovation Center of Traditional Chinese Medicine in Prevention and Treatment of Tumor, Nanjing, China

**Keywords:** colorectal cancer, extracellular matrix remodeling, gut microbiota, immunotherapy, traditional Chinese medicine, tumor immune microenvironment

## Abstract

Colorectal cancer (CRC) is a common malignancy of the digestive tract, and its initiation and progression are closely associated with dysregulation of the tumor immune microenvironment. The CRC immune microenvironment consists of effector immune cells, immunosuppressive cells, inflammatory mediators, immune checkpoint pathways, stromal cells, the extracellular matrix, the gut microbiota, and microbial metabolites. Its pathological features include impaired immune recognition and effector immunity, enhanced immunosuppression, extracellular matrix remodeling-mediated immune exclusion, dysregulated inflammatory and immune checkpoint signaling, and disturbances in gut microbiota and metabolism. Traditional Chinese medicine (TCM), characterized by multicomponent, multitarget, and multilevel regulation, has shown potential value in research on the CRC immune microenvironment. Current preclinical studies suggest that TCM-derived isolated compounds and bioactive constituents, single herbs, Chinese herbal formulas, and Chinese patent medicines may improve immune recognition and effector immune function by restoring antigen presentation, promoting dendritic cell maturation and enhancing effector T-cell responses and natural killer cell activity. These interventions may also inhibit immunosuppressive cells, modulate immune checkpoint and inflammation-related signaling, and enhance antitumor immunity by inducing immunogenic forms of tumor cell death. In addition, TCM may alleviate immunosuppression and immune exclusion by modulating stromal remodeling mediated by cancer-associated fibroblasts and regulating the gut microbiota, microbial metabolites, and tumor immunometabolism. In some preclinical models, these effects enhanced the antitumor efficacy of immune checkpoint blockade and provided experimental support for further mechanistic validation and clinical studies of TCM combined with immunotherapy. However, current evidence remains predominantly preclinical, and further clinical validation and assessment of bioavailability, safety, and drug interactions are needed. This review summarizes the composition and pathological features of the CRC immune microenvironment and discusses recent advances in TCM-mediated immune modulation. It also examines current mechanistic evidence and its limitations to inform further mechanistic validation, clinical study design, and translational research.

## 1 Introduction

Colorectal cancer (CRC) is one of the most common malignancies of the digestive tract, with approximately 1.926 million new cases worldwide and 517, 000 new cases in China in 2022, imposing a substantial disease burden (1, 2). The initiation and progression of CRC are driven not only by tumor cell-intrinsic alterations but also by continuous remodeling of the local immune microenvironment. The tumor immune microenvironment is composed of tumor cells, innate and adaptive immune cells, stromal cells, and the cytokines, chemokines, and metabolites released by these cellular components (3). The extent and spatial distribution of CD3 ^+^ and CD8 ^+^ T-cell infiltration in tumor tissues are closely associated with recurrence risk and clinical prognosis (4). During the malignant transformation of colorectal adenomas and subsequent metastatic progression, the composition and functional states of immune cells, as well as the interactions among tumor cells, immune cells, and stromal cells, undergo dynamic changes (5). Effective antigen presentation, together with the activation of effector T cells and natural killer (NK) cells, contributes to tumor immune clearance. In contrast, the accumulation of immunosuppressive cells, engagement of immune checkpoint pathways, and aberrant inflammatory signaling impair antitumor immunity and influence therapeutic responses across different molecular subtypes of CRC (3). Accordingly, modulation of the tumor immune microenvironment may help restore antitumor immune responses and alleviate immunosuppression, thereby providing a mechanistic rationale for developing new preventive and therapeutic strategies for CRC. In traditional Chinese medicine (TCM), CRC does not correspond to a specific classical disease entity. According to its clinical manifestations, it is commonly discussed within the categories of “intestinal accumulation” and “intestinal mass.” Although the primary disease site is the intestine, CRC is considered closely related to dysfunction of the spleen and stomach. Its core pathogenesis is characterized by the intermingling of dampness-heat and stasis-toxin, with spleen qi deficiency as the underlying root, thereby forming an accumulation disorder marked by deficiency in origin and excess in manifestation, as well as an intermingling of deficiency and excess. Spleen deficiency impairs the generation of qi and blood and weakens resistance to pathogenic factors, whereas prolonged accumulation of dampness-heat and stasis-toxin may further damage the intestinal tract, resulting in a pathological state of healthy qi deficiency combined with pathogenic excess. From the perspective of modern tumor immunology, healthy qi deficiency may be functionally related to impaired immune surveillance and weakened antitumor effector responses, whereas dampness-heat and stasis-toxin accumulation may be interpreted in association with chronic inflammation, immunosuppression, and intestinal microbial dysbiosis (3). Therefore, therapeutic strategies based on anticancer detoxification and strengthening healthy qi to consolidate the root can be conceptually linked to the restoration of effector immunity, attenuation of immunosuppression, and improvement of the local tumor microenvironment.

TCM is characterized by multiple components, multiple targets, and multilevel regulatory effects, which are compatible with the complexity of the CRC immune microenvironment. Recent studies on TCM-mediated regulation of the CRC immune microenvironment have increasingly extended to immune cells, immune checkpoint pathways, immunometabolism, and gut microecology. Nevertheless, the available evidence remains scattered across diverse therapeutic categories, immune regulatory processes, and mechanistic pathways, and therefore requires systematic integration. Previous reviews have mainly examined the conventional antitumor effects and related signaling pathways of TCM in CRC (6), the interactions among TCM, the gut microbiota, and CRC (7), or the potential sensitizing strategies of TCM against barriers to immune checkpoint blockade (ICB) in MSS CRC (8). These reviews have provided important references for understanding specific mechanisms of action or clinical therapeutic challenges. However, relatively few reviews have used the pathological organization of the CRC immune microenvironment as the central framework. Within such a framework, the effects of different levels of TCM interventions remain insufficiently integrated across immune recognition and effector responses, immunosuppression, stromal remodeling and immune exclusion, inflammatory and immune checkpoint signaling, and gut microbial and metabolic dysregulation. To complement this perspective, the present review integrates evidence on TCM-derived isolated compounds and bioactive constituents, single herbs, Chinese herbal formulas, and Chinese patent medicines. It also critically evaluates the available evidence in relation to disease stage, molecular subtype, model relevance, and translational limitations. Together, these analyses help clarify the potential roles of TCM in CRC prevention, treatment, and immune modulation.

## 2 Composition and pathological features of the immune microenvironment in colorectal cancer

**Figure 1:**
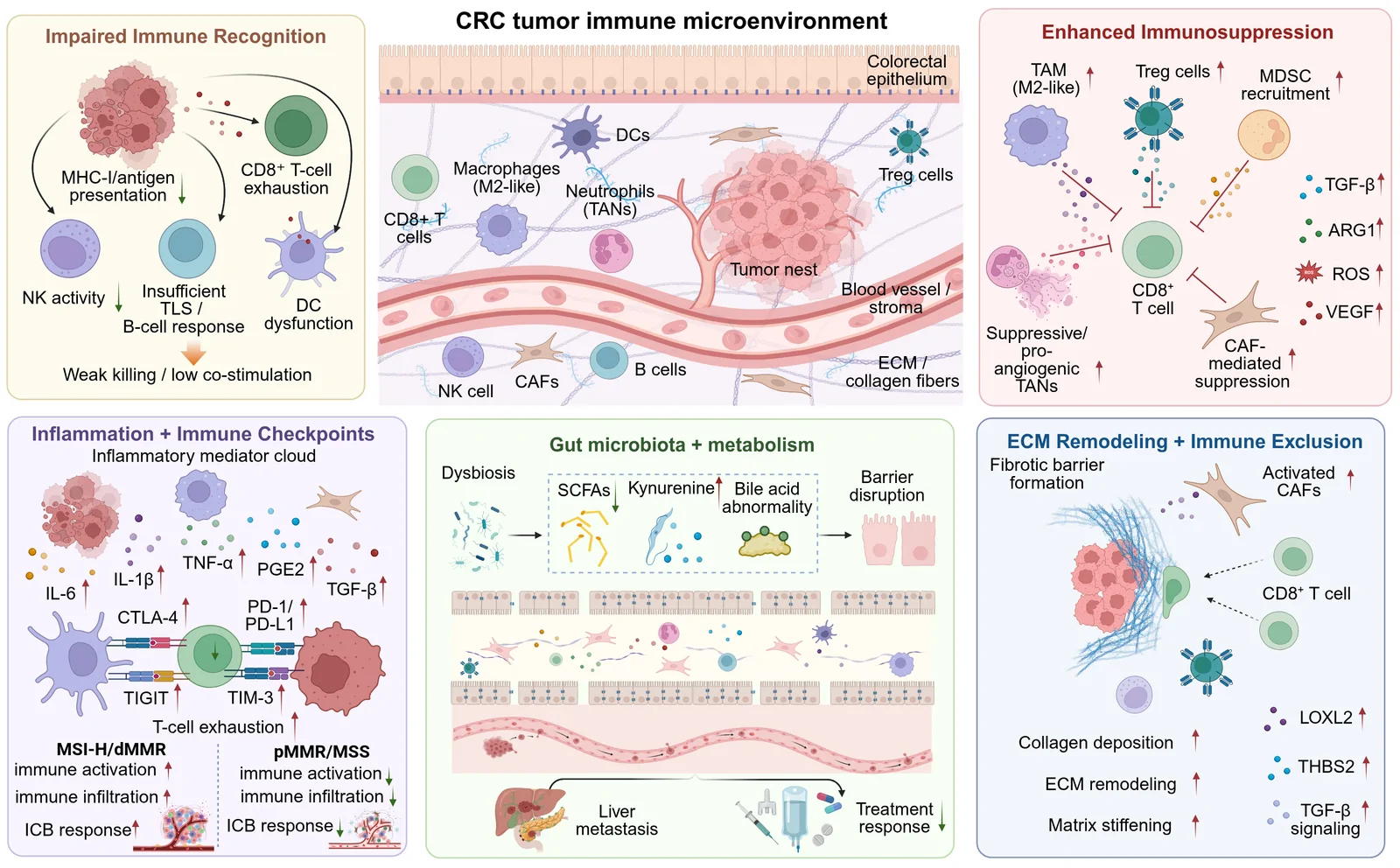
Pathological features of the CRC tumor immune microenvironment. Created in BioRender. Shi, Z. (2026) https://BioRender.com/bt65ssy.

The immune microenvironment of colorectal cancer is not determined by changes in any single immune cell population. Rather, it represents a dynamic network composed of effector immune cells, immunosuppressive cells, inflammatory mediators, immune checkpoint pathways, tumor stroma and extracellular matrix, the gut microbiota, and microbial metabolites. Given the distinct mismatch repair-deficient/microsatellite instability-high (dMMR/MSI-H) and mismatch repair-proficient/microsatellite-stable (pMMR/MSS) molecular subtypes, propensity for liver metastasis, and close involvement of gut microecology in CRC, immune microenvironmental dysregulation in CRC has features that are more specific to intestinal tumor biology. The pathological features of this microenvironment are mainly characterized by impaired antitumor immune responses, enrichment of immunosuppressive components, extracellular matrix remodeling-associated immune exclusion, aberrant inflammatory signaling, dysregulated immune checkpoint pathways, and disturbances in the gut microbiota and microbial metabolism. Together, these alterations constitute the pathological basis of CRC immune microenvironmental dysfunction and influence malignant transformation of colorectal adenomas, primary tumor formation, liver metastasis, and therapeutic response. Therefore, clarifying the composition and abnormal features of the CRC immune microenvironment is essential for understanding how TCM modulates immune states and antitumor responses in CRC. The pathological features of the CRC immune microenvironment are summarized in **Figure 1**.

### 2.1 Impaired immune recognition and effector responses

In the CRC immune microenvironment, CD3 ^+^ T cells and CD8 ^+^ T cells serve as key cellular indicators of antitumor immune activity. The level and spatial distribution of CD3 ^+^ and CD8 ^+^ T-cell infiltration in tumor tissues have been closely associated with patient prognosis, suggesting that effector T cells are important markers for evaluating the local immune status of CRC (9). However, the presence of effector T-cell infiltration does not necessarily translate into effective tumor killing. CD8 ^+^ T cells in CRC exhibit marked heterogeneity, encompassing tumor-reactive cytotoxic populations, bystander T cells, and functionally exhausted cell subsets. These distinct CD8 ^+^ T-cell states collectively shape heterogeneous immune microenvironmental profiles (10). Therefore, impaired effector immunity in CRC cannot be attributed solely to insufficient effector cell abundance, but is also closely associated with limited T-cell antigen specificity, functional exhaustion, and impaired coordination of local antitumor immune responses.

Insufficient immune recognition represents an upstream determinant of impaired effector immunity. CD8 ^+^ T-cell-mediated tumor killing requires the recognition of tumor antigens, whereas reduced MHC class I (MHC-I)-restricted antigen presentation compromises the ability of CD8 ^+^ T cells to recognize and eliminate tumor cells. SOX17-related studies suggest that colorectal adenomas and carcinomas may evade immune surveillance by disrupting IFN-γ-associated immune responses and antigen presentation, indicating that defective antigen presentation may occur across multiple stages of CRC initiation and progression (11). In parallel, dendritic cells (DCs), particularly subsets with cross-presenting capacity, are essential antigen-presenting cells required for initiating CD8 ^+^ T-cell-mediated antitumor responses. Defects in DC abundance or function may therefore restrict antitumor immunity. Notably, in liver metastases from pMMR CRC, the paucity of DCs has been identified as an important factor limiting the efficacy of ICB (12). Taken together, impaired antigen presentation by tumor cells and insufficient professional antigen-presenting cell support collectively underlie defective immune recognition and constrained effector T-cell responses in CRC.

In addition to CD8 ^+^ T cells, NK cells are important contributors to local effector immunity in CRC. Unlike CD8 ^+^ T cells, NK cells can mediate tumor cell killing independently of classical antigen presentation, primarily through perforin/granzyme-mediated cytotoxicity, and they can produce IFN-γ and other cytokines that support DC and T-cell responses. However, within the tumor microenvironment, hypoxia, lactate accumulation, and inhibitory checkpoint or receptor signals, including programmed cell death protein 1 (PD-1), programmed death-ligand 1 (PD-L1), and natural killer group 2A (NKG2A), may attenuate NK-cell activity, thereby compromising innate effector immunity (13).

In addition, unconventional effector lymphocytes, including γδ T cells, may also contribute to antitumor immunity in CRC. In HLA class I (HLA-I)-deficient tumors, γδ T cells can serve as immunotherapeutic effector cells, providing an alternative mechanism of immune recognition and cytotoxicity for tumors with defective antigen presentation (14). Tertiary lymphoid structures (TLSs) and B-cell-associated immune responses are also important components of local antitumor immunity in CRC. CCL19 ^+^ fibroblasts have been shown to promote TLS formation in CRC liver metastases and enhance antitumor IgG responses (15). Intratumoral CD8 ^+^CXCR5 ^+^ follicular-like cytotoxic T cells are associated with CD19 ^+^CD38 ^+^ B cells and TLSs and have prognostic relevance (16). These findings suggest that TLSs, B cells, and follicular-like cytotoxic T cells function as coordinated components of local effector immunity in CRC. Insufficient formation or functional immaturity of this local immune architecture may therefore compromise antitumor immune responses.

### 2.2 Enhanced immunosuppression

In addition to impaired immune recognition and effector dysfunction, enhanced immunosuppression represents another major feature of the dysregulated immune microenvironment in CRC. Single-cell and spatial omics studies have shown that, during colorectal tumorigenesis, the composition, functional states, and spatial interactions of immune cells undergo continuous remodeling. In this process, myeloid cells, regulatory T cells (Tregs), and stromal cells gradually establish a suppressive niche that favors tumor progression (17). Among these components, tumor-associated macrophages (TAMs) represent a key myeloid cell population involved in shaping the immunosuppressive microenvironment. Macrophage subsets with distinct spatial distributions and functional states are associated with different prognostic outcomes in patients with CRC, indicating that TAMs do not represent a homogeneous population but instead constitute a highly heterogeneous group of immune regulatory cells (18). In CRC liver metastases, SPP1 ^+^ macrophages can form an immunosuppressive spatial network with fibroblasts, thereby promoting the establishment of a metastatic microenvironment (19). Liver metastatic disease may also limit the efficacy of immunotherapy through macrophage-mediated T-cell elimination (20). These findings indicate that TAMs contribute not only to immunosuppression at the primary tumor site but also to immune evasion and therapeutic resistance in metastatic lesions through spatial cellular interactions and the elimination of effector T cells.

Myeloid-derived suppressor cells (MDSCs) are major contributors to impaired antitumor immunity. In CRC, MDSCs promote tumor progression by suppressing cytotoxic T lymphocyte activity, reshaping the inflammatory cytokine milieu, and facilitating immune evasion. DCLK1-related studies have shown that tumor cells can recruit MDSCs through the CXCL1-CXCR2 axis, thereby inhibiting tumor-specific cytotoxic T-cell responses (21). In addition, CXCR2 ^+^ MDSCs have been implicated in promoting colitis-associated tumorigenesis (22). These findings suggest that, during inflammation-associated colorectal tumorigenesis, MDSCs are not merely bystander immune infiltrates but constitute an important cellular link connecting chronic inflammation, immunosuppression, and tumor formation.

In addition to TAMs and MDSCs, Tregs, tumor-associated neutrophils (TANs), and cancer-associated fibroblasts (CAFs) also participate in the establishment of an immunosuppressive network. Spatial and single-cell colocalization analyses have shown that midkine (MDK)-related signaling can shape a Treg-associated immunosuppressive niche, suggesting that the function of Tregs is not limited to the secretion of inhibitory mediators such as IL-10 and transforming growth factor-β (TGF-β), but is also closely linked to their spatial localization and cellular interactions (23). TANs may undergo tissue-specific reprogramming into pro-angiogenic neutrophil states, thereby contributing to CRC vascularization and microenvironmental remodeling (24). In parallel, IL1R1 ^+^ CAFs have been shown to drive tumor progression and immunosuppression, indicating that stromal cells are not merely passive scaffolds but active participants in immune exclusion and the formation of an immunosuppressive microenvironment (25). Overall, enhanced immunosuppression in CRC is not driven by any single cell type alone. Rather, it results from spatially coordinated interactions among multiple cellular populations, including TAMs, MDSCs, Tregs, TANs, and CAFs, which collectively constrain effector immune responses and promote tumor progression.

### 2.3 Extracellular matrix remodeling and immune exclusion

The extracellular matrix (ECM) is a major structural component of the CRC tumor stroma and an important factor influencing immune cell infiltration and immunotherapeutic response. Studies on immuno-collagenic subtypes have shown that collagen deposition and immune infiltration jointly predict tumor responses to ICB. Tumors characterized by high collagen deposition and low immune infiltration are generally associated with poor responsiveness to immunotherapy (26). This evidence indicates that ECM and collagen-related structural features are not merely stromal alterations, but may also serve as important parameters for evaluating immune exclusion and immunotherapeutic response (27). Spatial omics and single-cell transcriptomic analyses in CRC patients further showed that the boundary between tumor and stroma differs in spatial organization and immune state between dMMR and pMMR CRC, and that these differences are associated with the efficacy of ICB. In responders, spatial interactions between LAMP3 ^+^ DCs and CXCL13 ^+^ T cells appear to contribute to an immune-activated boundary between tumor and stroma. In contrast, in non-responders, CXCL14 ^+^ CAFs tend to remodel the ECM and form structural barriers that restrict immune cell entry into tumor regions (28). These findings suggest that ECM-associated structural barriers may represent an important structural basis for the immune cold microenvironment and poor response to ICB in pMMR/MSS CRC.

CAFs are key cellular contributors to ECM remodeling in CRC. Fibroblast integrin α5 has been shown to be enriched in fibroblast activation protein-positive (FAP ^+^) CAFs and closely associated with ECM-related genes and collagen deposition. Genetic deletion or therapeutic targeting of α5 in fibroblasts reduced ECM deposition, increased intratumoral infiltration of CD8 ^+^IFN-γ ^+^ effector T cells, decreased Treg abundance, and potentiated PD-L1 blockade (29). Another study showed that an acidic microenvironment activated G protein-coupled receptor 4 (GPR4) in tumor cells. GPR4 then upregulated lysyl oxidase-like 2 (LOXL2) and TGF-β through Janus kinase 2/signal transducer and activator of transcription 3 (JAK2/STAT3) signaling, promoting type I collagen deposition and ordered collagen fiber alignment. These changes strengthened the barrier properties of the ECM, restricted CD8 ^+^ T-cell entry into tumor nests, and induced immune exclusion. Inhibition of LOXL2 or JAK2/STAT3 signaling increased intratumoral CD8 ^+^ T-cell infiltration and enhanced the response to PD-L1 blockade (30). Together, these studies indicate that ECM remodeling can restrict local antitumor immunity in CRC by altering collagen deposition, stromal architecture, and the spatial distribution of effector T cells, with contributions from both CAF-mediated matrix remodeling and tumor cell-driven collagen organization.

Fibrotic CRC is often characterized by an MSS phenotype and desmoplastic stroma, accompanied by insufficient immune infiltration. THBS2-expressing matrix CAFs can form an immune-exclusion barrier at the tumor front, thereby restricting CD8 ^+^ T-cell entry into tumor regions. Targeting THBS2 disrupts this barrier, promotes intratumoral CD8 ^+^ T-cell infiltration, and increases tumor sensitivity to ICB (31). In mesenchymal-type CRC, fibroblast TXNDC5 enhances TGF-β signaling by stabilizing TGFBR1, thereby promoting fibroblast activation, tumor stiffening, and fibrotic barrier formation. Fibroblast-specific deletion of Txndc5 reduces tumor stiffness, alleviates hypoxia, increases cytotoxic T-cell recruitment, and enhances the efficacy of PD-1 blockade (32). In addition, translational research based on immuno-collagenic subtypes suggests that AGTR1 is highly expressed in CAFs within tumors characterized by high collagen deposition and low immune infiltration. Angiotensin receptor blockers can enhance the efficacy of ICB by suppressing type I collagen expression in CAFs (33). Therefore, ECM remodeling constitutes a structural component of the immunosuppressive network in CRC. By promoting collagen deposition and fibrotic barrier formation and by restricting effector T-cell entry, ECM remodeling can act together with cellular immunosuppressive factors such as TAMs, MDSCs, and Tregs to promote an immune-refractory microenvironment.

### 2.4 Inflammatory signaling and immune checkpoint dysregulation

Dysregulation of inflammatory mediators, immune checkpoint pathways, and related signaling cascades represents an important manifestation of CRC immune microenvironmental imbalance and provides a mechanistic basis for differences in immunotherapeutic response among CRC molecular subtypes. dMMR/MSI-H tumors generally harbor a higher tumor mutational burden and increased neoantigen load owing to defective mismatch repair and are often accompanied by more pronounced intratumoral T-cell infiltration. Consequently, these tumors are more likely to respond to ICB. In contrast, pMMR/MSS tumors commonly display insufficient immune infiltration, limited effector T-cell activation, and poor responsiveness to immunotherapy (34). Clinical studies have shown that PD-1 blockade and combined blockade of PD-1 and CTLA-4 confer substantial therapeutic benefits in locally advanced dMMR rectal cancer, dMMR colon cancer, and MSI-H metastatic CRC (35–37). These findings indicate that differences in response to ICB cannot be explained solely by checkpoint molecules themselves, but also reflect heterogeneity in tumor immunogenicity and microenvironmental immune activation across CRC molecular subtypes.

The effects of PD-1 blockade are not limited to the restoration of T-cell function. In dMMR CRC, PD-1 blockade has been shown to alter the composition of immune and stromal cell populations within tumor tissues, suggesting that immunotherapeutic responses involve broad remodeling of the multicellular network in the tumor microenvironment (38). However, not all patients with CRC benefit from ICB. This limitation is especially relevant in pMMR/MSS CRC, where inflammatory mediators and immunosuppressive pathways often restrict the expansion and cytotoxic function of effector T cells. Prostaglandin E2 (PGE2) can limit the expansion of tumor-infiltrating stem-like CD8 ^+^ T cells into effector cells, thereby impairing antitumor immunity (39). Moreover, TAM-derived PGE2 can promote CD8 ^+^ T-cell exhaustion and drive resistance to PD-L1 blockade (40). Collectively, inflammatory mediators and immunosuppressive myeloid cells shape the microenvironmental basis of poor immunotherapeutic responsiveness in pMMR/MSS CRC by influencing T-cell differentiation, expansion, and exhaustion.

Inflammation-related signaling may further amplify local immunosuppression through stromal and myeloid cell compartments. Interleukin-6/interleukin-11 (IL-6/IL-11)-mediated activation of STAT3 can act on CAFs and promote colorectal tumor progression (41). A TGF-β-activated tumor microenvironment can induce macrophage polarization toward an M2-like phenotype, accompanied by increased expression of T-cell immunoglobulin and mucin-domain containing-3 (TIM-3), thereby reinforcing local immunosuppression (42). In addition, SOX17-mediated early immune evasion suggests that tumor cell-intrinsic transcriptional programs may influence the microenvironment by interfering with IFN-γ-associated immune responses, antigen presentation, and immune recognition (11). Therefore, dysregulation of the CRC immune microenvironment cannot be simply attributed to reduced immune cell abundance or checkpoint upregulation. Rather, it should be understood as an imbalance in a signaling network involving tumor cells, immune cells, stromal cells, inflammatory mediators, and immune checkpoint pathways.

### 2.5 Gut microbiota and metabolic dysregulation

The gut microbiota represents a distinctive and particularly relevant determinant of the CRC immune microenvironment compared with many non-intestinal solid tumors. Studies integrating tumor, immune, and microbiome profiles have shown that the microbial composition of colon cancer tissues is closely associated with tumor immune status, with distinct microbial communities corresponding to different patterns of immune infiltration, inflammatory states, and tumor biological features (43). Accordingly, the gut microbiota should not be regarded merely as a commensal microbial community colonizing the intestinal lumen. Rather, it may contribute to the shaping of the CRC immune microenvironment by affecting intestinal barrier integrity, local inflammatory responses, metabolite profiles, and immune cell activation states. In addition, disruption of the gut vascular barrier has been shown to promote the dissemination of intestinal bacteria and facilitate CRC liver metastasis, suggesting that microbial dysbiosis may contribute to metastatic progression through barrier disruption and remodeling of immune ecosystems in distant organs (44).

Certain cancer-associated microbial taxa may promote CRC progression through both tumor cell-intrinsic alterations and immune modulation. *Escherichia coli* carrying the pks island can induce characteristic mutational signatures in CRC, indicating that specific microbiota-derived genotoxic factors may directly compromise tumor genomic stability (45). *Fusobacterium nucleatum* can upregulate PD-L1 expression through ALPK1 activation, thereby facilitating tumor immune evasion (46). In addition, *F. nucleatum* can downregulate MLH1 expression by activating the autophagy-lysosome pathway, suggesting that the microbiota may influence MMR-associated molecular status and the context of immunotherapeutic response (47). These findings indicate that the role of cancer-associated microbiota in CRC is not limited to inflammation induction. Instead, these microbial factors may contribute to immune microenvironmental dysregulation through DNA damage, immune checkpoint modulation, and altered expression of DNA repair-related molecules.

In contrast to pro-tumorigenic microbial taxa, certain beneficial bacteria and their metabolites can enhance antitumor immunity. Butyrate produced by *Roseburia intestinalis* can activate cytotoxic CD8 ^+^ T cells and thereby enhance the efficacy of PD-1 blockade (48). *Bifidobacterium catenulatum* has also been shown to increase the sensitivity of MSS CRC to PD-1 blockade by activating CD8 ^+^ T cells (49). In addition, *Clostridium butyricum* can improve the efficacy of PD-1 blockade by suppressing IL-6-mediated immunosuppression (50). These findings indicate that the gut microbiota does not merely influence tumor cell growth. Rather, it contributes to the dynamic regulation of the CRC immune microenvironment by modulating CD8 ^+^ T-cell activation, inflammatory cytokine production, and the immunosuppressive state of the CRC immune microenvironment.

Microbiota-derived metabolites serve as important mediators linking the gut microbiota to the immune microenvironment, among which short-chain fatty acids (SCFAs) and bile acids have received particular attention. Microbiota-derived bile acids can activate Takeda G protein-coupled receptor 5 (TGR5) and promote CRC liver metastasis by inducing hepatic MDSC infiltration (51). In MSS CRC, *Bacteroides fragilis* (*B. fragilis*) and propionate have also been shown to influence responses to radiotherapy and immunotherapy (52). These findings indicate that dysregulation of the gut microbiota and microbial metabolites contributes not only to primary CRC development, immune evasion, and liver metastasis formation, but also to therapeutic responsiveness. Given that TCM interventions often exert multitarget regulatory effects on gut microbiota, inflammatory responses, and the immune microenvironment, modulation of the gut microbiota and its metabolites represents an important mechanistic perspective for understanding how TCM regulates the CRC immune microenvironment.

## 3 Regulatory effects of isolated compounds and single herbs

TCM-derived isolated compounds and defined bioactive constituents generally have relatively well-defined material bases and traceable mechanisms of action, whereas single herbs retain greater multicomponent complexity. Together, they provide complementary levels of evidence for elucidating how TCM may regulate the CRC immune microenvironment. Preclinical and mechanistic studies suggest that their reported antitumor activities are not limited to the direct inhibition of tumor cell proliferation and migration or the induction of apoptosis. Instead, these activities may involve multiple immunological processes, including antigen presentation, activation of effector immune cells, modulation of immunosuppressive myeloid cells, regulation of immune checkpoint pathways, induction of immunogenic cell death, CAF-associated stromal signaling and gut microbiota-mediated immune modulation. Although different classes of bioactive constituents exhibit distinct mechanistic preferences, they appear to converge on modulating the imbalance among tumor immune recognition, effector immunity, and immunosuppressive states in experimental settings.

### 3.1 TCM-derived isolated compounds and bioactive constituents

#### 3.1.1 Flavonoids

Baicalin is one of the major flavonoid glycosides derived from the dried root of *Scutellaria baicalensis*. In preclinical CRC models, baicalin inhibited Toll-like receptor 4/nuclear factor-κB (TLR4/NF-κB) signaling, downregulated PD-L1 expression and the frequency of MDSCs, and increased CD4 ^+^ and CD8 ^+^ T-cell infiltration, thereby enhancing local antitumor immune activity (53). In addition, baicalin promoted the proteasomal degradation of hexokinase 2 (HK2), inhibited glycolysis, and activated cyclic GMP-AMP synthase/stimulator of interferon genes (cGAS/STING)-IFN-β signaling, which in turn promoted the polarization of TAMs toward an M1-like phenotype (54). These preclinical findings suggest that baicalin may modulate the CRC immune microenvironment by affecting PD-L1-mediated immunosuppression, MDSC accumulation, effector T-cell infiltration, and TAM polarization. Both studies used subcutaneous syngeneic tumor models, so whether these effects also occur in orthotopic, metastatic, or human CRC remains unclear.

In the cited Rg3-quercetin co-delivery study, quercetin mainly enhanced immunogenic cell death induced by Rg3 in CRC cells. On the basis of Rg3-induced immunogenic cell death in CRC models, quercetin further enhanced Rg3-mediated immunogenic cell death by promoting reactive oxygen species (ROS) production, thereby amplifying immunostimulatory signals released during tumor cell death (55). These preclinical findings suggest that quercetin may function as an enhancer of Rg3-mediated immunogenic cell death and facilitate the connection among tumor cell death, antigen presentation, and subsequent antitumor immune responses. More recently, quercetin enhanced CD8 ^+^ T-cell effector function and reduced metastatic burden in an experimental CRC lung metastasis model by targeting BCAT1 and regulating branched-chain amino acid metabolism (56). These findings extend the evidence beyond the co-delivery system, although the immune effects reported in the earlier *in vivo* study cannot be attributed to quercetin alone and the available evidence remains preclinical.

Epimedii Folium possesses anti-inflammatory and antitumor pharmacological activities, and preclinical evidence suggests that its bioactive constituents and metabolites may exert anti-CRC effects by modulating the tumor immune microenvironment. In coculture and mouse CRC models, icariin inhibited M2-like macrophage polarization through the PI3K/Akt signaling pathway, thereby attenuating TAM-associated immunosuppression and suppressing CRC progression (57). Icariin also promoted ferroptosis in CRC cells by inducing mitochondrial dysfunction and enhanced the antitumor activity of PD-1 blockade in preclinical CRC models (58). In addition, Icaritin inhibited tumor growth and prolonged survival in the MC38 colorectal tumor model. However, its immunomodulatory effects and sensitization to PD-1/CTLA-4 blockade were mainly demonstrated in the B16F10 melanoma model, limiting their direct relevance to CRC (59). These preclinical findings suggest that Epimedii Folium and its related flavonoid constituents may participate in the regulation of the CRC immune microenvironment by suppressing TAM- and MDSC-mediated immunosuppression, promoting effector T-cell infiltration, and increasing responsiveness to ICB in experimental models. Ferroptosis inhibition in the icariin study and CD8 ^+^ T-cell depletion experiments strengthened the causal link between immune activation and tumor control in the latter two studies. However, findings for icariin and icaritin should not be extended directly to Epimedii Folium as a whole, and the checkpoint blockade combinations have not been tested in patients with CRC.

The clinical translation of flavonoids is limited by low oral exposure and complex metabolism. The bioavailability of quercetin in humans varies with its glycoside form and formulation (60). After oral administration, baicalin and icariin can be converted into metabolites such as baicalein and icaritin, respectively (61, 62). Therefore, the parent compounds and concentrations used *in vitro* may not reflect the active forms present *in vivo*. Most CRC studies have not measured parent compounds and metabolites in plasma and tumor tissues at the same time. The relationship between drug exposure and immune effects also remains unclear. Future studies should combine pharmacokinetics, tumor distribution, and immune endpoints to determine whether effective exposure for regulating the CRC immune microenvironment can be achieved in humans.

#### 3.1.2 Saponins

Cycloastragenol, an active small molecule derived from *Astragalus membranaceus* root, is a cycloartane-type triterpene aglycone (63). The cited preclinical study showed that cycloastragenol targets cathepsin B, reduces lysosomal degradation of MHC-I, and promotes MHC-I accumulation on the tumor cell surface, thereby enhancing tumor antigen presentation. In subcutaneous tumor-bearing models and CRC organoid experiments, cycloastragenol treatment enhanced CD8 ^+^ T-cell-mediated antitumor responses and suppressed tumor growth. Moreover, combined treatment with cycloastragenol and anti-PD-1 antibody further increased antitumor activity in these preclinical models (63). These preclinical findings suggest that cycloastragenol may regulate the CRC immune microenvironment mainly by restoring tumor antigen presentation and enhancing CD8 ^+^ T-cell-mediated immune clearance. The study established a relatively complete mechanistic link between cathepsin B inhibition, MHC-I preservation, and CD8 ^+^ T-cell function. However, independent validation remains limited, and the subcutaneous tumor and organoid systems do not fully reproduce the immune and stromal complexity of human CRC.

Astragaloside IV (ASIV) is a representative triterpenoid saponin derived from Astragali Radix. In preclinical CRC models, ASIV inhibited the biogenesis and release of extracellular vesicles (EVs) from CRC cells and downregulated EV-related molecules, including nSMase2 and Rab27a. Conditioned medium from ASIV-treated CRC cells reduced M2-like macrophage polarization and promoted M1-like macrophage polarization, thereby weakening the invasive and migratory capacities of CRC cells. In a spleen-to-liver metastasis model, ASIV also reduced macrophage and M2-like macrophage infiltration in liver metastatic lesions and suppressed metastasis formation (64). A recent follow-up study identified TERT as a direct target of ASIV and linked reduced exosome biogenesis to regulation of the RALY/PLD2 axis in the same metastasis setting (65). In addition, in an azoxymethane/dextran sulfate sodium (AOM/DSS)-induced colitis-associated CRC model, ASIV alleviated intestinal inflammation, decreased IL-6, TNF-α, IL-1β, and iNOS, activated PPARγ-Nrf2/HO-1 signaling, and reduced oxidative stress-related DNA damage, thereby inhibiting inflammation-associated tumorigenesis in mice (66). These preclinical findings suggest that ASIV may modulate the metastatic immune microenvironment by blocking tumor-derived EV-mediated M2-like polarization of TAMs, while also attenuating immune microenvironmental dysregulation in inflammation-associated CRC through the modulation of PPARγ-related inflammatory and oxidative stress signaling. The follow-up study further clarified how ASIV suppresses EV production, while additional studies are needed to determine how EV regulation, TAM responses, epithelial inflammation, and oxidative DNA damage together influence the CRC immune microenvironment.

Ginsenoside Rg3 (Rg3) is a naturally occurring saponin derived from *Panax ginseng*. In a preclinical Rg3 and quercetin co-delivery study, Rg3 induced immunogenic cell death in CRC cells and promoted DC maturation (55). *In vitro* and orthotopic xenograft studies showed that Rg3 inhibited CRC cell growth and migration, reduced the expression of angiogenesis-related genes, and suppressed tumor vascularization. It can also downregulate B7-H1, also known as PD-L1, and B7-H3, suggesting that Rg3 may influence the tumor microenvironment by inhibiting angiogenesis and attenuating immunosuppressive checkpoint-related signals in preclinical models (67). A recent oral nanoplatform that co-delivered Rg3 and ursolic acid increased immunogenic cell death, DC maturation, and tumor-infiltrating lymphocytes and enhanced PD-1 blockade in an MSS CRC mouse model (68). These preclinical findings suggest that Rg3 may influence the CRC immune microenvironment by enhancing tumor immunogenicity and regulating checkpoint-associated molecules. Current immune evidence for Rg3 comes mainly from combination and delivery systems, which makes its independent contribution difficult to define. More broadly, saponins may regulate the CRC immune microenvironment, but their *in vivo* conversion remains a concern. Animal pharmacokinetic studies show that orally administered Astragalus saponins circulate mainly as cycloastragenol metabolites. Exposure to unchanged ASIV is low, whereas direct cycloastragenol administration results in faster absorption and higher systemic exposure (69). Rg3 also has low oral bioavailability, rapid clearance, and microbiota-dependent metabolism. Its stereochemistry and formulation further affect its pharmacokinetic behavior (70). Few CRC studies have measured parent compounds and metabolites in plasma and tumor tissues at the same time, and the relationship between drug exposure and immune effects remains unclear. Future studies should identify the active forms and exposure levels that mediate immune regulation within tumors.

#### 3.1.3 Phenolic compounds

Curcumin is a major natural polyphenolic constituent derived from the rhizome of *Curcuma longa* and is one of the principal bioactive components of turmeric (71). In preclinical pMMR/MSS CRC models, curcumin was reported to upregulate gasdermin E (GSDME) by inhibiting the ubiquitin-proteasome system (UPS) and to activate the caspase-3/GSDME axis, thereby inducing tumor cell pyroptosis. In CT26 tumors, curcumin-enhanced pyroptosis increased the infiltration of CD45 ^+^ immune cells and CD3 ^+^, CD4 ^+^, and CD8 ^+^ T cells, reduced the proportion of Foxp3 ^+^CD4 ^+^ Tregs, and potentiated the efficacy of PD-1 blockade (72). In another mouse CRC study, curcumin modulated gut microbiota composition, promoted intratumoral CD8 ^+^ T-cell infiltration, increased the levels of effector cytokines, including IFN-γ, TNF-α, and IL-2, and exerted antitumor effects partly through the induction of ferroptosis. Fecal microbiota transplantation (FMT) and gut microbiota depletion experiments further indicated that its antitumor activity is at least partly microbiota-dependent in this model (73). A further experimental study using CT26-derived CAFs and a BALB/c nude mouse colon cancer model showed that curcumin inhibited CAF proliferation and TGF-β secretion and indirectly downregulated PD-L1 expression in CRC cells through CAF modulation. In the same model, curcumin combined with PD-1 blockade increased M1-like macrophages and CD8 ^+^ T cells while reducing M2-like macrophages and Tregs, thereby enhancing antitumor immune responses (74). Collectively, the available preclinical evidence supports a potential role for curcumin in modulating the CRC immune microenvironment by inducing immunogenic forms of tumor cell death, enhancing effector T-cell infiltration, attenuating Treg-mediated immunosuppression, regulating the gut microbiota, and interfering with CAF-associated suppressive signaling. Further studies should identify the microbial factors involved and confirm the CAF-related immune effects in immunocompetent CRC models.

In experimental CRC models, the combination of honokiol, magnolol, and baicalin enhanced tumor immunogenicity mainly through GSDME-dependent pyroptosis and increased the antitumor activity of PD-1 blockade (75). Together with the findings on curcumin, these experimental studies suggest that phenolic compounds may modulate the CRC immune microenvironment by inducing immunogenic forms of tumor cell death, promoting effector T-cell infiltration, and increasing responsiveness to ICB in preclinical settings. In addition, certain phenolic compounds, particularly curcumin, may further modulate the gut microbiota as well as inflammatory and metabolic states, thereby contributing to immune microenvironmental remodeling in CRC models. Comparisons of single and combined treatments support the synergistic activity of honokiol, magnolol, and baicalin, although the contribution of each component to pyroptosis and immune regulation remains unclear.

Evidence remains limited on whether phenolic compounds can achieve sufficient and sustained exposure *in vivo* to support their preclinical immune effects. Studies show that plasma concentrations of free curcumin remain low even with absorption-enhancing formulations. These concentrations are also far below those commonly used *in vitro*. Measuring only total curcumin or conjugated metabolites may overestimate its bioavailable exposure (76). Honokiol and magnolol also have limited solubility and delivery efficiency. Nanocarriers may improve their delivery and tumor accumulation, but relevant evidence in CRC remains scarce (77).

#### 3.1.4 Other compounds

Bufalin is a representative bufadienolide derived from Chansu, also known as toad venom. In metastatic CRC models, highly metastatic tumor cells promoted M2-like TAM polarization through Krüppel-like factor 4 (KLF4) and increased PD-L1 expression on TAMs, thereby suppressing cytotoxic T-cell function and facilitating immune escape. Bufalin targeted steroid receptor coactivator-3 (SRC-3), reduced KLF4 release, and decreased M2-like TAM polarization and PD-L1 expression, thereby attenuating the immunosuppressive microenvironment (78). Another experimental study showed that CAF-conditioned medium promoted CRC cell migration, invasion, and epithelial-mesenchymal transition (EMT), accompanied by increased p-STAT3, MMP-2, and MMP-9 expression and decreased E-cadherin expression. Bufalin inhibited CAF-mediated STAT3 activation and EMT-related changes, reduced the invasive and metastatic capacity of CRC cells, and decreased metastatic lesion formation in a liver metastasis model (79). A bufalin-loaded phototherapeutic Janus membrane induced immunogenic cell death, promoted DC maturation, increased CD8 ^+^ T cells and NK cells, reduced MDSCs, and downregulated PD-L1 in a preclinical postoperative CRC model (80). The immune response resulted from bufalin, local delivery, and phototherapy, so the contribution of bufalin alone remains difficult to define. In preclinical oxaliplatin-resistant CRC models, bufalin reduced SRC-3-dependent macrophage migration inhibitory factor (MIF) release from resistant tumor cells, suppressed M2-like macrophage polarization, and enhanced oxaliplatin activity (81). A recent study extended these findings to hypoxic CRC models, where bufalin inhibited SRC-3/hypoxia-inducible factor-1α (HIF-1α) signaling, reduced M2-like macrophage polarization, and reversed oxaliplatin resistance (82). Together, these macrophage studies identify SRC-3 in tumor cells as a recurring regulator of immunosuppressive signaling, with KLF4, MIF, and HIF-1α involved in different disease settings. Overall, current preclinical evidence suggests that bufalin may regulate the CRC immune microenvironment by disrupting communication between tumor cells and macrophages, while delivery systems may further induce immunogenic cell death and enhance DC and cytotoxic lymphocyte responses.

The use of bufalin requires careful consideration of cardiotoxicity and interactions among multiple constituents. Recent experimental studies show that bufalin can impair mitochondrial energy metabolism and induce excessive autophagy, resulting in dose-dependent cardiotoxicity (83). The combined use of multiple bufadienolides in Chansu can also alter their pharmacokinetic profiles. This finding suggests that potential drug interactions should be considered when bufalin-containing preparations are combined with other anticancer agents (84). Clinical studies suggest that cinobufacini combined with oxaliplatin-based chemotherapy may improve short-term efficacy, peripheral CD4 ^+^ T-cell levels, and some adverse events. However, the available randomized trials remain limited in quality, and these effects cannot be attributed directly to bufalin (85). Future studies should define an exposure range that can regulate the CRC immune microenvironment without causing significant cardiac injury.

Active triterpenoid fractions derived from *Rhus chinensis* Mill. may improve effector T-cell function by regulating immunometabolism. In the cited preclinical study, these fractions reversed effector CD8 ^+^ T-cell dysfunction, enhanced their antitumor activity, and inhibited CRC growth by improving glycolytic metabolism (86). A subsequent mouse study further reported increased intratumoral CD8 ^+^ T-cell accumulation and regulation of PD-1 and PD-L1 expression (87). Because the tested material was a multicomponent triterpenoid fraction, the constituents responsible for these immune effects remain to be identified. Together, these experimental findings indicate that different active constituents may regulate the CRC immune microenvironment through complementary mechanisms, including suppression of immunosuppressive cellular networks and restoration of effector immunity.

Although the triterpenoid fraction has been characterized by high-performance liquid chromatography (HPLC), it remains a multicomponent extract. The main active constituents and their pharmacokinetic profiles in plasma and tumor tissues have not been identified (86). Low doses of aqueous and ethanol extracts from Rhus chinensis Mill. fruits caused no obvious adverse effects, whereas high doses induced injury to the heart, liver, spleen, and kidneys (88). Future studies should define a safe dose range that can improve CD8 ^+^ T-cell function in CRC. Representative TCM-derived isolated compounds and bioactive constituents involved in CRC immune microenvironment regulation are summarized in **Table 1**.

**Table 1:** Key mechanisms of TCM-derived isolated compounds and bioactive constituents in modulating the CRC immune microenvironment.

Component(s)	Main immune target(s)	Key mechanism(s)	Potential application	Model system(s)	MMR/MSI status	Dose/exposure	Preclinical evidence level
Baicalin	MDSCs, T cells, TAMs	TLR4/NF-κB, PD-L1; HK2–cGAS/STING	Relieve myeloid immunosuppression	CRC cells; CT26 and MC38 subcutaneous tumors	NR	20–40 μM; 20–40 mg/kg	P2
Quercetin	Tumor immunogenicity, CD8 ^+^ T cells	ROS–ICD; BCAT1/BCAA metabolism	Enhance ICD and T-cell function	CRC cells; CT26 orthotopic tumors; MC38 lung metastasis	NR	10 μM; 4–10 mg/kg	P2
Icariin and icaritin	TAMs, MDSCs, CD8 ^+^ T cells	PI3K/Akt; ferroptosis; PD-1 sensitization	Sensitize to PD-1 blockade	Macrophage coculture; AOM/DSS; CT26 and MC38 subcutaneous tumors	NR	10–20 μM; 70–100 mg/kg	P3
Cycloastragenol	Antigen presentation, CD8 ^+^ T cells	CTSB inhibition; reduced MHC-I degradation	Restore antigen presentation	CRC cells and organoids; CT26 and MC38 subcutaneous tumors	NR	12.5 μM; 50 mg/kg	P2
Astragaloside IV	TAMs, inflammatory microenvironment	EVs–nSMase2/Rab27a, RALY/PLD2; PPARγ–Nrf2/HO-1	Suppress metastasis and inflammation	Coculture; spleen-to-liver metastasis; AOM/DSS	NR	12.5 μM; 20–80 mg/kg	P3
Ginsenoside Rg3	ICD, DCs, checkpoint molecules	ICD, DC maturation; PD-L1, B7-H3	Enhance tumor immunogenicity	CRC cells; CT26 orthotopic tumors; xenografts	MSS in one study; others NR	10–25 mg/kg	P1
Curcumin	T cells, Tregs, microbiota, CAFs	GSDME-mediated pyroptosis, ferroptosis, microbiota modulation; CAF/TGF-β/PD-L1	Sensitize to PD-1 blockade	CRC cells; CAF coculture; CT26 subcutaneous and orthotopic tumors; nude-mouse model	pMMR/MSS in one study; others NR	5–10 μM; 10–30 mg/kg	P3
Honokiol, magnolol, and baicalin	Tumor immunogenicity, T cells	GSDME-dependent pyroptosis	Sensitize to PD-1 blockade	CRC cells; HCT116 xenografts; MC38 orthotopic tumors	NR	10/60/100 mg/kg, respectively	P1
Bufalin	TAMs, CAFs, DCs, CD8 ^+^ T and NK cells	SRC-3/KLF4, CAF/STAT3, SRC-3/MIF/HIF-1α; ICD	Target metastasis and resistance	Coculture; subcutaneous, orthotopic, liver-metastasis, drug-resistant, and postoperative models	NR	0.5–1 mg/kg; local sustained release	P3
Triterpenoid fractions from *Rhus chinensis* Mill.	CD8 ^+^ T cells, PD-1/PD-L1	Glycolysis and immunometabolism	Restore effector T-cell function	CT26 cells; CT26 subcutaneous tumors	NR	25 mg/kg	P2

MMR/MSI status was recorded only when explicitly reported; NR denotes not reported, with no inference from model characteristics. Dose/exposure indicates representative *in vitro* concentrations and *in vivo* doses. For this review, P1 denotes preliminary or combination-dependent evidence; P2, mechanistically supported evidence across experimental systems; and P3, consistent evidence from multiple studies and models. These levels reflect preclinical mechanistic evidence, not clinical efficacy.

### 3.2 Single herbs in traditional Chinese medicine

#### 3.2.1 Heat-clearing and toxin-resolving herbs

*Salvia plebeia* is a heat-clearing and toxin-resolving herb that is traditionally used to cool the blood and promote diuresis. It has been reported to possess anti-inflammatory, antitumor, and immunomodulatory activities. In biochemical and cell-based assays, *Salvia plebeia* ethanol extract and its constituent cosmosiin inhibited PD-1/PD-L1 binding and reactivated T-cell receptor (TCR) signaling. In humanized PD-1 knock-in mice bearing human PD-L1-expressing MC38 tumors, oral administration of Salvia plebeia extract increased intratumoral CD3 ^+^ and CD8 ^+^ T-cell infiltration and inhibited tumor growth (89). These effects connect the inhibition of abnormal immune checkpoint signaling with the recovery of effector T-cell activity in the CRC immune microenvironment. The humanized model strengthens this mechanistic link, although the *in vivo* effects were demonstrated for the whole extract rather than cosmosiin alone.

Marsdeniae Tenacissimae Caulis (Tongguanteng) is traditionally used to clear heat, resolve toxins, relieve cough, and calm wheezing. In a preoperative observational study, 17 patients with stage II or III CRC received oral Marsdenia tenacissima tablets for two weeks. Increased CD3 ^+^ and CD8 ^+^ T-cell infiltration was observed in paired tumor samples from 13 of these patients (90). In mechanistic experiments, *Marsdenia tenacissima* extract reduced TGF-β1 and PD-L1 expression in HCT116 and LoVo CRC cells. In Jurkat T cells cultured alone or with CRC cells, the extract decreased FOXP3 and IL-10 expression and increased IL-2 expression. In AOM/DSS-induced and CT26 subcutaneous tumor models, Marsdenia tenacissima treatment increased intratumoral CD3 ^+^ and CD8 ^+^ T-cell infiltration and reduced tumor burden (90). By reducing TGF-β1/PD-L1-mediated inhibitory signaling and Treg-related immunosuppressive features, Marsdenia tenacissima may favor the recovery of intratumoral effector T-cell responses in CRC. Nevertheless, the available human evidence is limited to a small, uncontrolled preoperative cohort with short-term biomarker assessment and does not establish improved clinical outcomes.

#### 3.2.2 Blood-cooling herbs

Sanguisorbae Radix is traditionally used to cool the blood, stop bleeding, resolve toxins, and promote wound healing, and is commonly applied in conditions such as gastrointestinal bleeding. In competitive binding and cell-based reporter assays, Sanguisorbae Radix ethanol extract inhibited the PD-1/PD-L1 interaction and increased TCR/nuclear factor of activated T cells (NFAT) signaling activity. In coculture experiments, the extract increased IL-2 release from humanized PD-1 splenocytes and perforin 1 (PRF1) release from tumor-derived CD8 ^+^ T cells and enhanced T-cell-mediated killing of human PD-L1-expressing MC38 cells (91). In a humanized PD-L1-expressing CRC mouse model, Sanguisorbae Radix extract suppressed tumor growth. Its combination with pembrolizumab further increased intratumoral CD8 ^+^ T-cell infiltration and enhanced their cytotoxic activity (91). Through PD-1/PD-L1 blockade, Sanguisorbae Radix may alleviate checkpoint-mediated immunosuppression and restore CD8 ^+^ T-cell infiltration and cytotoxic activity within the CRC immune microenvironment. The humanized checkpoint model provides targeted mechanistic support, although the findings remain limited to a multicomponent extract in preclinical systems and the active constituents responsible for PD-1/PD-L1 blockade have not been identified.

Lithospermi Radix (Zicao) is traditionally used to clear heat, cool the blood, promote blood circulation, and resolve toxins. Its representative naphthoquinone constituent, shikonin, has been reported to exert antitumor activity. In CT26 cells, shikonin promoted cell-surface calreticulin exposure and upregulated heat shock protein 70 (Hsp70) through pyruvate kinase M2 (PKM2) -dependent ROS production, thereby inducing immunogenic features of tumor cell death. When combined with PD-1 blockade, shikonin increased intratumoral DC and CD8 ^+^ T-cell infiltration in CT26 tumor-bearing mice and enhanced antitumor efficacy (92). By increasing tumor cell immunogenicity, shikonin may promote effector T-cell infiltration within the CRC immune microenvironment and improve the responsiveness of MSS tumors to PD-1 blockade. PKM2 silencing and ROS inhibition supported the upstream mechanism, but whether Hsp70 is required for the enhanced *in vivo* immune response was not directly tested.

#### 3.2.3 Blood-activating and stasis-resolving herbs

Saffron (Croci Stigma) is classified as a blood-activating and stasis-resolving herb, with additional traditional functions of cooling the blood, resolving toxins, relieving constraint, and calming the mind. *In vitro* experiments showed that saffron ethanolic extract, crocin, and safranal inhibited the proliferation of CRC cells. In CT26 subcutaneous tumor-bearing mice, crocin and safranal reduced serum IL-17 levels, downregulated IL-6, TNF-α, TGF-β, and PD-L1 expression in tumor tissues, and modulated splenic CD4 ^+^ and CD8 ^+^ T-cell populations and their ratio (93). Crocin and safranal may therefore improve the CRC immune microenvironment by suppressing IL-17-associated inflammatory signaling, reducing tumor PD-L1 expression, and modulating systemic T-cell responses. Because orally administered crocin is extensively converted by the gut microbiota to crocetin, which reaches much higher systemic exposure, future CRC studies should determine whether this metabolite contributes to the immune effects attributed to crocin (94).

*Salvia miltiorrhiza* Bunge is a commonly used blood-activating and stasis-resolving herb in clinical practice. Traditionally, it is used to activate blood circulation, dispel blood stasis, unblock the meridians, relieve pain, clear the heart, alleviate restlessness, cool the blood, and resolve abscesses. In an MC38 subcutaneous tumor model, its aqueous extract inhibited the cyclooxygenase-2 (COX-2)/PGE2 cascade, reduced TAM infiltration, increased the proportions of IFN-γ ^+^ and GZMB ^+^ CD8 ^+^ T cells, and enhanced the antitumor effect of PD-L1 blockade (95). In an MC38 lung-metastasis model, rosmarinic acid combined with ginsenoside Rg1 cooperatively interfered with COX-2 and PD-1/PD-L1-related signaling and suppressed lung metastasis of MC38 colon cancer (96). These preclinical findings suggest that *Salvia miltiorrhiza* aqueous extract may modulate the CRC immune microenvironment mainly by limiting TAM accumulation and supporting CD8 ^+^ T-cell activity, whereas the rosmarinic acid–ginsenoside Rg1 combination links suppression of COX-2-dependent metastatic behavior with PD-1/PD-L1-related immune regulation. Because the metastatic study evaluated rosmarinic acid in combination with ginsenoside Rg1, the observed effects cannot be attributed to Salvia miltiorrhiza or rosmarinic acid alone.

#### 3.2.4 Other herbs

Chinese yam (Dioscoreae Rhizoma) is a medicinal and edible herb traditionally used to tonify the spleen, nourish the stomach, promote fluid generation, benefit the lung, tonify the kidney, and secure essence. It has been reported to possess immunomodulatory and antitumor activities. In MC38 and CT26 subcutaneous tumor models, Chinese yam polysaccharide enhanced the tumor-suppressive effect of an anti-PD-1 monoclonal antibody. This effect was dependent on the gut microbiota and was accompanied by changes in microbial metabolites, increased intratumoral CD8 ^+^ T-cell infiltration and granzyme B expression, and reduced CD206 ^+^ M2-like macrophages (97). These findings suggest that Chinese yam polysaccharide may improve the CRC immune microenvironment and enhance the response to PD-1 blockade by reducing microbiota-associated deoxyguanosine, limiting CD206 ^+^ M2-like macrophage polarization, and increasing CD8 ^+^ T-cell infiltration and granzyme B expression. Although deoxyguanosine promoted M2-like macrophage polarization and weakened the efficacy of PD-1 blockade, whether restoring this metabolite can reverse the benefit of the Chinese yam polysaccharide combined with PD-1 blockade was not directly tested.

Cortex Periplocae (Xiangjiapi) is a wind-dampness-dispelling herb traditionally used to dispel wind-dampness, strengthen the tendons and bones, and promote urination to reduce swelling. In DSS-induced colitis and AOM/DSS-induced colitis-associated CRC models, Cortex Periplocae reshaped gut microbiota composition and suppressed pathogenic T helper 17 (Th17) cell responses, thereby alleviating colitis and limiting the development of colitis-associated CRC (98). These preclinical findings suggest that Cortex Periplocae may help maintain intestinal mucosal immune homeostasis and ameliorate microbiota-associated immune dysregulation during colitis-associated CRC through normalization of gut microbial composition and restraint of pathogenic Th17 responses. Representative single herbs and their major immune microenvironment-related mechanisms in CRC are summarized in **Table 2**.

**Table 2:** Key mechanisms of single herbs in modulating the CRC immune microenvironment.

Single herb	Main immune target(s)	Key mechanism(s)	Potential application	Model system(s)	MMR/MSI status	Dose/exposure	Preclinical evidence level
Salvia plebeia	PD-1/PD-L1, CD8 ^+^ T cells	PD-1/PD-L1 blockade; TCR/NFAT reactivation	Relieve T-cell dysfunction	Binding and coculture assays; humanized PD-1/hPD-L1 MC38 tumors	NR	0–50 μg/mL; 100–300 mg/kg	P2
Marsdeniae Tenacissimae Caulis	Tregs, effector T cells	Reduced TGF-β1/PD-L1 and FOXP3/IL-10; increased IL-2	Restore effector T-cell immunity	17-patient paired tumor samples; CRC–T-cell coculture; AOM/DSS; CT26 subcutaneous tumors	NR	2.4 g, three times daily, 2 weeks; 5–20 mL/kg	Exploratory clinical
Sanguisorbae Radix	PD-1/PD-L1, CD8 ^+^ T cells	PD-1/PD-L1 blockade; TCR/NFAT and IL-2/PRF1 activation	Sensitize to PD-1 blockade	Binding and coculture assays; humanized PD-1/hPD-L1 MC38 tumors	MSI	0–50 μg/mL; 100–300 mg/kg	P2
Lithospermi Radix	DCs, CD8 ^+^ T cells, tumor immunogenicity	Shikonin–PKM2–ROS–Hsp70 axis	Sensitize to PD-1 blockade	CT26 cells; CT26 subcutaneous tumors	MSS	5–10 μM; 3 mg/kg	P2
Saffron	Inflammatory cytokines, T cells, PD-L1	IL-17-associated inflammation; reduced IL-6/TNF-α/TGF-β/PD-L1	Relieve inflammatory immunosuppression	CT26 and HCT116 cells; CT26 subcutaneous tumors	NR	Crocin, 10–30 mg/kg; safranal, 200–600 mg/kg	P1
Salvia miltiorrhiza Bunge	TAMs, CD8 ^+^ T cells, PD-L1	COX-2/PGE2; COX-2–MYO10 and PD-1/PD-L1	Sensitize to PD-L1 blockade	MC38 cells; MC38 subcutaneous and lung-metastasis models	NR	Aqueous extract, 5–10 g/kg; rosmarinic acid/Rg1, 100 mg/kg/day each	P2
Chinese yam	Gut microbiota, CD8 ^+^ T cells, M2-like macrophages	Microbiota and metabolite modulation; reduced deoxyguanosine; suppressed M2 polarization	Sensitize to PD-1 blockade	MC38 and CT26 subcutaneous tumors; microbiota depletion and metabolite validation	NR	Chinese yam polysaccharide, 100 mg/kg	P2
Cortex Periplocae	Gut microbiota, Th17 cells	Microbiota remodeling; pathogenic Th17 suppression	Maintain mucosal immune homeostasis	DSS and AOM/DSS models; microbiota depletion/FMT; CD4 ^+^ T-cell depletion	NR	Periploca sepium periplosides, 6.25–12.5 mg/kg	P2

MMR/MSI status was recorded only when explicitly reported; NR denotes not reported, without inference from model characteristics. Dose/exposure lists representative *in vitro* concentrations, *in vivo* doses, or human dosing. P1 denotes preliminary or combination-dependent evidence; P2, mechanistically supported evidence across experimental systems; and P3, consistent evidence across multiple studies and models. Exploratory clinical denotes a small, uncontrolled study with short-term immune biomarkers and does not establish clinical efficacy. P1–P3 reflect preclinical mechanistic support only.

In summary, the available evidence is derived predominantly from cell-based and animal studies. These findings suggest that TCM-derived isolated compounds, bioactive constituents, and single herbs may modulate the CRC immune microenvironment through multiple mechanisms. These mechanisms include enhancement of antigen presentation and effector T-cell function, regulation of immune checkpoint pathways and inflammatory signaling, suppression of immunosuppressive myeloid cells, modulation of CAF-associated stromal signaling, and regulation of the gut microbiota. TCM-derived isolated compounds are useful for identifying relatively well-defined active substances and specific regulatory processes, whereas single herbs further reflect the multicomponent and multitarget regulatory characteristics of TCM. On this basis, studies on Chinese herbal formulas and Chinese patent medicines may better capture the holistic features of TCM-mediated intervention in the CRC immune microenvironment.

## 4 Regulatory effects of Chinese herbal formulas and Chinese patent medicines

Chinese herbal formulas can act on immune cells, inflammatory responses, immunometabolism, and the gut microbiota through coordinated multicomponent and multitarget effects, thereby reflecting the holistic regulatory characteristics of TCM in modulating the CRC immune microenvironment. Chinese patent medicines, with fixed prescriptions, defined dosage forms, and relatively stable quality control, provide a more standardized pharmacological basis for mechanistic validation and clinical application. In addition, certain experimental preparations and modern delivery systems have expanded the application modalities of TCM-derived bioactive constituents in regulating the tumor immune microenvironment.

### 4.1 Chinese herbal formulas

#### 4.1.1 Classical formulas

Wumei Wan, first recorded in the *Treatise on Cold Damage* (*Shang Han Lun*), is composed of Mume Fructus, Asari Radix et Rhizoma, Zingiberis Rhizoma, Coptidis Rhizoma, Angelicae Sinensis Radix, Aconiti Lateralis Radix Praeparata, Zanthoxyli Pericarpium, Cinnamomi Ramulus, Ginseng Radix et Rhizoma, and Phellodendri Chinensis Cortex. Traditionally, it is used to harmonize cold and heat, support healthy qi, and eliminate pathogenic factors. In an AOM/DSS-induced colitis-associated CRC mouse model, Wumei Wan inhibited indoleamine 2, 3-dioxygenase 1 (IDO1) expression and tryptophan-to-kynurenine metabolism, thereby regulating the IDO1-kynurenine (KYN)-aryl hydrocarbon receptor (AhR) signaling axis and attenuating KYN metabolism-mediated immunosuppression (99). During the early stage of AOM/DSS-induced colitis-associated CRC tumorigenesis in mice, Wumei Wan also regulated amino acid metabolism and PI3K/Akt signaling, reduced MDSC accumulation, and weakened MDSC-mediated immunosuppressive function (100). These findings suggest that Wumei Wan may modulate the immune microenvironment in colitis-associated CRC models by correcting abnormal tryptophan and amino acid metabolism, suppressing MDSC-mediated myeloid immunosuppression and supporting antitumor T-cell responses.

Sijunzi Decoction, composed of Ginseng Radix et Rhizoma, Atractylodis Macrocephalae Rhizoma, Poria, and Glycyrrhizae Radix et Rhizoma, is a representative formula for tonifying qi and strengthening the spleen. In a subcutaneous colon cancer mouse model and NK-cell coculture experiments, Sijunzi Decoction modulated p53 expression, upregulated death receptors DR4 and DR5 in tumor cells, and increased tumor-cell sensitivity to NK-cell-mediated cytotoxicity (101). In a nude-mouse colon cancer liver metastasis model, the modified Sijunzi Decoction increased plasma granulocyte-macrophage colony-stimulating factor (GM-CSF) levels and splenic macrophage numbers, while reducing liver metastasis formation (102). Collectively, these findings suggest that Sijunzi Decoction may support innate antitumor immunity in experimental CRC models by increasing tumor-cell susceptibility to NK-cell killing and modulating macrophage-associated responses. The NK-cell mechanism was supported by depletion and p53 inhibition experiments, whereas the macrophage findings were based mainly on changes in cell numbers and GM-CSF levels.

Huang-Lian-Jie-Du Decoction (HLJDD), composed of Coptidis Rhizoma, Scutellariae Radix, Phellodendri Chinensis Cortex, and Gardeniae Fructus, is traditionally used to purge fire and resolve toxins. Among CRC patients receiving capecitabine and oxaliplatin, higher baseline fecal Akkermansia muciniphila abundance was associated with a better treatment response. In MC38 and CT26 tumor-bearing mice, HLJDD combined with capecitabine and oxaliplatin increased A. muciniphila abundance, intratumoral CD8 ^+^ T-cell infiltration, and IFN-γ expression, and produced greater tumor growth inhibition than chemotherapy alone (103). These findings suggest that HLJDD may improve the CRC immune microenvironment through A. muciniphila-associated changes in tryptophan metabolism and CD8 ^+^ T-cell responses, thereby strengthening the antitumor effect of chemotherapy in these mouse models. The patient findings support *A. muciniphila* as a potential marker of XELOX response, but the chemosensitizing effect of HLJDD itself has so far been demonstrated only in mouse models.

Banxia Xiexin Decoction, first recorded in the *Treatise on Cold Damage* (*Shang Han Lun*), is traditionally used to harmonize cold and heat, relieve focal distension, and dissipate masses. In an AOM/DSS-induced colitis-associated colorectal cancer (CAC) rat model, Banxia Xiexin Decoction reduced tumor formation, inhibited STAT3-related inflammatory signaling, and ameliorated gut microbial dysbiosis, thereby attenuating chronic inflammation-driven tumor progression (104). In a separate AOM/DSS-induced CAC mouse model, Banxia Xiexin Decoction decreased the levels of inflammatory cytokines, including TNF-α, IL-6, IL-1β, and IFN-γ, and improved the inflammation-associated microenvironment by regulating glycerophospholipid metabolism and pathways related to the complement and coagulation cascades (105). These findings suggest that Banxia Xiexin Decoction may ameliorate microbiota- and inflammation-associated immune dysregulation during experimental CAC development by modulating the gut microbiota, suppressing STAT3-mediated inflammatory responses, and influencing complement-related immune and inflammatory pathways. A study involving 80 patients with stage III colon cancer reported that modified Banxia Xiexin Decoction improved quality of life and prolonged disease-free survival when combined with chemotherapy (106). Another randomized trial involving 129 patients with advanced CRC found improvements in several tumor markers after six treatment cycles, but no significant prolongation of progression-free or overall survival (107). Neither study assessed changes in the gut microbiota or immune cells, and direct clinical evidence that Banxia Xiexin Decoction modulates the CRC immune microenvironment therefore remains limited.

Tong-Xie-Yao-Fang (TXYF), first recorded in the *Danxi Xinfa*, is traditionally used to tonify the spleen, soothe the liver, dispel dampness, and arrest diarrhea. Chronic stress can exacerbate immunosuppression and promote tumor progression in CRC. In a chronic restraint stress-associated CRC mouse model, TXYF promoted DC maturation and enhanced antigen-presenting capacity, increased the proportion of CD4 ^+^ T cells and the CD4 ^+^/CD8 ^+^ T-cell ratio, shifted the Th1/Th2 balance toward Th1, upregulated IFN-γ, IL-18, IL-2, and IL-12, and decreased IL-4 and IL-10, thereby enhancing T-cell-mediated antitumor immune responses (108). In addition, in an AOM/DSS-induced colitis-associated CRC mouse model, TXYF induced mitophagy in colonic epithelial cells through the PTEN-induced kinase 1 (PINK1)/Parkin pathway, thereby suppressing inflammation-associated tumorigenesis and reversing EMT (109). These findings suggest that TXYF may participate in regulating the immune microenvironment in experimental CRC models by activating the DC–T-cell axis, correcting chronic stress-associated immunosuppression, and interfering with inflammation-related epithelial injury and carcinogenic progression. However, these immune effects have only been demonstrated in mouse models, and their relevance to the immune microenvironment of CRC patients remains unclear.

Shenling Baizhu San (SLBZS), first recorded in the *Taiping Huimin Heji Jufang*, is traditionally used to tonify qi, strengthen the spleen, drain dampness, and arrest diarrhea. In a BALB/c-Hpd1 mouse xenograft model established with HCT116 cells, SLBZS combined with tislelizumab produced greater tumor growth inhibition than tislelizumab alone. The combination also altered gut microbiota composition, increased circulating M1-like macrophages, and reduced circulating M2-like macrophages, MDSCs, and Tregs (110). In addition, in an MC38-Luc liver metastasis model established by intrasplenic injection, SLBZS combined with PD-1 blockade reduced metastatic burden. Its active constituent neferine inhibited cytochrome P450 2E1 (CYP2E1), regulated CYP2E1-peroxisome proliferator-activated receptor alpha (PPARα)-mediated lipid metabolism, reduced homovanillic acid (HVA) accumulation, suppressed Treg expansion and CD8 ^+^ T-cell exhaustion, and thereby restored effector T-cell function and strengthened the response to PD-1 blockade in this model (111). An earlier AOM/DSS study also showed that SLBZS reduced colonic MDSC infiltration and TGF-β1 expression while suppressing EMT during colitis-associated CRC development (112). These findings suggest that SLBZS may regulate the CRC immune microenvironment and enhance the response to PD-1 blockade by modifying the gut microbiota, reducing myeloid- and Treg-associated immunosuppression, restoring CD8 ^+^ T-cell function, and correcting lipid metabolic abnormalities in liver metastases. The xenograft study assessed immune cells in peripheral blood, whereas the liver metastasis study examined the local immune microenvironment; whether SLBZS can improve PD-1 blockade in CRC patients remains unknown.

#### 4.1.2 Modern empirical formulas

Xiaoai Jiedu Recipe (XAJDR) is an empirical anticancer formula established by Professor Zhongying Zhou, a National TCM Master. XAJDR inhibited colon tumor growth in CT26 tumor-bearing mice, reduced the proportion of CD4 ^+^Foxp3 ^+^ Tregs in peripheral blood and the spleen, and decreased Foxp3 expression in tumor tissues, suggesting that it may improve the CRC immune microenvironment by attenuating Treg-mediated immunosuppression (113). XAJDR-medicated serum enhanced NK-cell-mediated cytotoxicity against HCT-116 cells, activated the STAT4-IFN-γ pathway, and promoted tumor cell apoptosis by downregulating Bcl-2 and Bcl-xL and upregulating Bax (114). Cell and xenograft experiments further showed that XAJDR inhibited CRC growth and altered gut microbiota composition and serum metabolite profiles, suggesting that microbiota-associated metabolic changes may contribute to its regulation of the CRC microenvironment (115). Taken together, these cell-based and mouse studies suggest that XAJDR may regulate the CRC immune microenvironment by reducing Treg-mediated immunosuppression, enhancing NK cell cytotoxicity, and modulating gut microbiota-related metabolic networks. The Treg findings were obtained in mice, whereas the NK-cell effect was shown only *in vitro* and the microbiota study did not assess immune-cell changes.

Xianlian Jiedu Decoction (XLJDD) is a modern empirical formula developed by Professor Haibo Cheng for the prevention and treatment of CRC recurrence and metastasis. It is traditionally used to clear heat, resolve dampness, exert anticancer and toxin-resolving effects, and strengthen the spleen while replenishing qi. Evidence from AOM/DSS-treated mice showed that XLJDD altered gut microbiota composition and metabolite profiles, reduced inflammatory cytokine levels, and improved gut microecological status (116). Transcriptomic, proteomic, and single-cell analyses of AOM/DSS-induced colorectal tumors indicated that XLJDD regulated potential targets such as Mfsd2a and Ccdc85c and altered the cellular composition of the tumor microenvironment, including reduced B-cell infiltration (117). However, because the study reported a change in total B-cell infiltration without resolving functional B-cell subsets, the immunological significance of this decrease remains unclear. In an orthotopic CRC mouse model, XLJDD reduced neutrophil infiltration in tumor tissues, downregulated peptidylarginine deiminase 4 (PAD4), citrullinated histone H3 (H3cit), and neutrophil elastase (NE), inhibited neutrophil extracellular trap (NET) formation, and attenuated EMT and liver metastasis (118). A separate mouse study of CRC liver metastasis found that XLJDD suppressed Plaur/Hbegf/EGFR-related signaling and cytoskeleton-remodeling effectors, including Fscn1 and Cdc42ep1, reduced Plaur-high malignant cell subpopulations, and weakened Spp1-Cd44 communication between malignant cells and THBS1 ^+^ TAMs, thereby inhibiting the formation of liver metastatic lesions (119). Collectively, these findings suggest that XLJDD may regulate the CRC immune microenvironment by improving gut microecology, altering B-cell infiltration, suppressing neutrophil-mediated NET formation, and weakening pro-metastatic communication between malignant cells and TAMs. These studies are strengthened by the use of several CRC models, multi-omics and single-cell analyses, and functional validation across both tumor development and liver metastasis.

Shen-Bai-Jie-Du Decoction (SBJDD) is a modern empirical formula developed by Professor Haibo Cheng for preventing the progression of colorectal adenoma to carcinoma. Single-cell analyses of Apc^Min/+^ mice and human colorectal specimens identified the progressive accumulation of CCL22 ^+^ immunosuppressive DCs and their close association with Tregs during colorectal tumorigenesis. SBJDD treatment reduced CCL22 ^+^ DCs in Apc^Min/+^ mice and in paired adenoma samples from three patients. Mechanistic experiments further indicated that SBJDD inhibited CCL22 ^+^ DC differentiation through TMEM131-TNF signaling, thereby attenuating DC-Treg network-mediated immunosuppression (120). Among patients with colorectal adenomas, SBJDD administration altered fecal microbiota composition and increased several fecal and serum SCFAs. Complementary experiments in Apc^Min/+^ mice showed that SBJDD increased SCFA production, decreased IL-1β and IL-6 expression, increased IL-10 expression, and attenuated inflammation during adenoma progression (121). The human sample analyses and mechanistic studies suggest that SBJDD may improve the immune microenvironment during colorectal adenoma progression by inhibiting CCL22 ^+^ DC differentiation, weakening immunosuppressive interactions between DCs and Tregs, and regulating gut microbiota, SCFA production, and inflammation.

Jianpi Jiedu formula (JPJDF) is traditionally used to tonify qi, strengthen the spleen, clear heat, and resolve toxins. Evidence from AOM/DSS-treated mice showed that JPJDF regulated tryptophan metabolism and suppressed KYN-AhR signaling, thereby reducing M2-like TAM polarization and attenuating TAM-associated immunosuppression (122). A CRC liver-metastasis study combining mouse and cell-based experiments showed that JPJDF reduced ITGBL1 levels in tumor-derived EVs and inhibited fibroblast activation and the expression of IL-6, IL-8, and α-SMA through TNFAIP3-NF-κB signaling, thereby weakening the CAF-driven inflammatory metastatic microenvironment (123). A separate murine liver-metastasis study found that JPJDF suppressed tumor cell glutamine uptake and metabolism through the YTHDF1/GID8-SLC1A3/GLS axis. This was accompanied by reduced macrophage infiltration and increased T cells and mature DCs in metastatic liver lesions (124). Collectively, these animal and mechanistic studies suggest that JPJDF may regulate the immune microenvironment of primary and metastatic CRC by modulating tryptophan and glutamine metabolism, reducing TAM- and CAF-associated immunosuppression, and increasing T-cell and mature DC abundance in liver metastatic lesions. In a randomized double-blind trial of 137 patients with metastatic CRC, JPJDF combined with fluoropyrimidine maintenance extended median progression-free survival from 3 to 5 months and median overall survival from 9 to 15 months, without increasing common adverse reactions (125). These clinical findings support the translational potential of JPJDF, although immune and stromal changes were not assessed in patients.

Qizhen Decoction, composed of prepared Astragali Radix, Ligustri Lucidi Fructus, Codonopsis Radix, Hedyotidis Diffusae Herba, Gynostemmae Pentaphylli Herba, and other herbs, is traditionally used to support healthy qi, activate blood circulation, resolve blood stasis, clear heat, and resolve toxins. In AOM/DSS-induced colitis-associated CRC mice, Qizhen Decoction increased gut microbial abundance, shifted gut microbiota composition toward that of healthy controls, reduced the dominance of pro-inflammatory and tumor-promoting bacteria such as *Bacteroides*, and increased beneficial bacteria, including *Bifidobacterium*, *Bacillus*, *Lactococcus*, and *Lactobacillus*. These changes were accompanied by alleviation of colonic inflammation and dysplasia (126). Using conventional and antibiotic-treated microbiota-depleted CRC mice, the study showed that Qizhen Decoction increased Akkermansia abundance, promoted DC maturation and IL-12 secretion, activated JAK2/STAT4 signaling, and enhanced effector T-cell responses. Qizhen Decoction combined with PD-1 blockade produced a stronger antitumor effect than PD-1 blockade alone in these mice (127). These findings suggest that Qizhen Decoction may improve the CRC immune microenvironment by increasing Akkermansia abundance, promoting DC maturation and IL-12 secretion, and enhancing effector T-cell responses, thereby strengthening the response to PD-1 blockade in these mouse models. The use of antibiotic-treated microbiota-depleted mice strengthens the evidence for a microbiota-dependent immune mechanism, although this effect has not yet been evaluated in CRC patients.

Jiedu Sangen Decoction (JSD) is an empirical formula developed according to the therapeutic principle of clearing heat and resolving toxins for the treatment of gastrointestinal malignancies. Medicated-serum coculture experiments involving CT26 cells and CAFs showed that JSD reduced tumor-cell migration, upregulated sirtuin 1 (SIRT1) expression in CAFs, decreased phosphorylated p65 expression, and inhibited NF-κB pathway activation in CAFs. These cell-based findings suggest that JSD may attenuate the pro-invasive and pro-metastatic effects of CAFs by modulating CAF-associated inflammatory stromal signaling (128). The coculture model directly captured tumor cell–CAF interactions, although this stromal effect has not yet been confirmed *in vivo*. In addition, JSD inhibited CRC cell proliferation and promoted apoptosis. Potential bioactive components, including resveratrol, genistein, and afzelin, together with candidate targets such as carbonic anhydrase 9 (CA9), NOTUM, and dipeptidase 1 (DPEP1), provide further mechanistic support for its anti-CRC activity (129). Overall, the available cell-based evidence suggests that JSD may influence CAF-related stromal processes relevant to the CRC immune microenvironment; however, direct immune remodeling and *in vivo* efficacy have not yet been established. Representative Chinese herbal formulas and their key mechanisms in modulating the CRC immune microenvironment are summarized in **Table 3**.

**Table 3:** Key mechanisms of Chinese herbal formulas in modulating the CRC immune microenvironment.

Chinese herbal formula	Main immune target(s)	Key mechanism(s)	Potential application	Model system(s)	MMR/MSI	Dose/exposure	Evidence tier
Wumei Wan	MDSCs, Th17/Treg, CD8 ^+^ T cells	IDO1–KYN–AhR; PI3K/Akt; amino acid metabolism	Relieve immunosuppression in CAC	AOM/DSS CAC mice; multi-omics	NR	6–54 g/kg/day	Tier 2
Sijunzi Decoction	NK cells, macrophages	p53–DR4/DR5; GM-CSF	Enhance innate antitumor immunity	Coculture; subcutaneous and liver metastasis models	NR	45 g/kg; medicated serum	Tier 2
Huang-Lian-Jie-Du Decoction	Gut microbiota, CD8 ^+^ T cells	*A. muciniphila*; tryptophan metabolism; IFN-γ	Enhance XELOX efficacy	MC38 and CT26 models; patient fecal analysis	NR	100–200 mg/kg/day	Tier 2
Banxia Xiexin Decoction	Gut microbiota, inflammatory mediators	STAT3; cytokines; complement/coagulation	Improve the CAC inflammatory milieu	AOM/DSS models; CRC clinical studies	NR	0.95–15.66 g/kg/day	Tier 2
Tong-Xie-Yao-Fang	DCs, T cells	DC maturation; Th1/Th2 balance; PINK1/Parkin	Relieve stress-related immunosuppression	Stress-associated CRC and AOM/DSS models; cells	NR	6.825–19.5 g/kg/day; 5%–10% serum	Tier 2
Shenling Baizhu San	TAMs, MDSCs, Tregs, CD8 ^+^ T cells	Microbiota remodeling; M1/M2 regulation; CYP2E1–PPARα	Enhance PD-1 blockade	Subcutaneous, liver metastasis, and AOM/DSS models	NR	0.5–14.56 g/kg/day	Tier 2
Xiaoai Jiedu Recipe	Tregs, NK cells, gut microbiota	Foxp3; STAT4–IFN-γ; metabolic regulation	Suppress Tregs and enhance NK activity	CT26 and HCT116 models; coculture	NR	5–20 g/kg; medicated serum	Tier 2
Xianlian Jiedu Decoction	B cells, TANs, NETs, TAMs	NET formation; Plaur/Hbegf/EGFR; immune-cell communication	Prevent recurrence and liver metastasis	Orthotopic and liver metastasis models; single-cell analysis	NR	1.95–25.8 g/kg/day	Tier 2
Shen-Bai-Jie-Du Decoction	CCL22 ^+^ DCs, Tregs, gut microbiota	TMEM131–TNF; SCFA regulation	Inhibit adenoma progression	Mouse models; single-cell and human sample analyses	NR	14.3 g/kg/day; 94 g/day in patients	Tier 3
Jianpi Jiedu Formula	TAMs, CAFs, T cells, DCs	KYN–AhR; ITGBL1-rich EVs; glutamine metabolism	Remodel primary and metastatic microenvironments	CAC, subcutaneous, and liver metastasis models	NR	4.17–24 g/kg/day; 50–200 μg/mL	Tier 2
Qizhen Decoction	Gut microbiota, DCs, T cells	DC maturation; IL-12/JAK2/STAT4	Enhance PD-1 response	CAC and tumor-bearing mouse models	NR	2.52 g/kg/day	Tier 2
Jiedu Sangen Decoction	CAFs, stromal signaling	Sirt1–NF-κB	Suppress pro-invasive stroma	CT26-conditioned fibroblast model	NR	10% medicated serum	Tier 1

MMR/MSI status was recorded only when explicitly reported; NR denotes not reported. Dose/exposure lists representative regimens and is not directly comparable across preparations. Evidence tiers indicate the highest source directly supporting the listed immune mechanism: Tier 1, *in vitro*/in silico; Tier 2, CRC or CAC animal models; and Tier 3, immune evaluation after intervention in patients or paired human samples. Clinical use, observational outcomes, or ex vivo human models without intervention-linked human immune assessment do not qualify as Tier 3. **Tables 3**–**4** use source-based tiers because evidence for formulas and formulated products is more heterogeneous than the P1–P3 mechanistic grading used in **Tables 1**–**2**.

### 4.2 Chinese patent medicines and granule preparations

Zhenqi Fuzheng Granule (ZQFZG), composed of Astragali Radix and Ligustri Lucidi Fructus, is a clinically approved Chinese patent medicine used to strengthen healthy qi. An earlier Apc^Min/+^ mouse study also showed that ZQFZG increased circulating T and NK cells, increased CD4 and CD8 expression in colorectal tumors, and reduced tumor CTLA-4 and PD-L1 expression (130). AOM/DSS-induced CRC mice treated with ZQFZG showed alterations in the gut microbiota and increased levels of SCFAs, particularly butyrate. Activation of G protein-coupled receptor 109A (GPR109A) was accompanied by inhibition of Akt/mTOR/HIF-1α signaling, tumor glycolysis, and lactate production (131). The same mouse study further showed that ZQFZG reduced MDSC accumulation, promoted macrophage polarization toward an M1-like phenotype, and restored the CD4 ^+^/CD8 ^+^ T-cell ratio. Its combination with an anti-PD-1 antibody produced greater tumor suppression than either treatment alone (131). FMT from ZQFZG-treated donors into antibiotic-pretreated mice further suppressed tumor growth, downregulated glycolysis-related proteins, including PKM2, glucose transporter 1 (GLUT1), HIF-1α, and lactate dehydrogenase A (LDHA), reduced MDSCs, and increased the infiltration of M1-like macrophages and effector T cells (131). The integration of multi-omics analyses, FMT, and butyrate intervention strengthens the evidence that microbial changes contribute to the immunometabolic effects of ZQFZG. These animal and mechanistic experiments suggest that ZQFZG may improve the CRC immune microenvironment by regulating the gut microbiota and SCFA production, suppressing tumor glycolysis and lactate accumulation, reducing MDSC-mediated immunosuppression, promoting M1-like macrophage polarization, and supporting effector lymphocyte responses.

Huaier granule is a Chinese patent medicine prepared from the aqueous extract of the medicinal fungus *Trametes robiniophila* Murr. and is clinically used as an adjuvant therapy for cancer. A propensity score-matched analysis of CRC patients found that Huaier use was associated with longer overall survival. Mechanistic experiments using CRC cells, AOM/DSS-induced mice, and MC38 subcutaneous tumors showed that Huaier activated STAT1-MHC-I signaling, increased MHC-I antigen-presentation components, promoted intratumoral CD8 ^+^ T-cell infiltration and cytolytic activity, and reduced Treg accumulation. Huaier also enhanced T-cell-mediated tumor killing when combined with PD-1 blockade in patient-derived organoids and produced greater tumor suppression than either monotherapy in MC38-bearing mice (132). The combination of patient survival data, patient-derived organoids, and immunocompetent mouse models strengthens the translational relevance of the study, although the survival analysis remains observational. In these experimental systems, Huaier may improve the CRC immune microenvironment by restoring MHC-I-dependent antigen presentation, enhancing CD8 ^+^ T-cell-mediated immune recognition, and reducing Treg accumulation, thereby strengthening the response to PD-1 blockade.

Qingjie Fuzheng Granule (QJFG) is composed of Hedyotidis Diffusae Herba, Scutellariae Barbatae Herba, Astragali Radix, Hordei Fructus Germinatus, and other Chinese medicinal herbs. In an AOM/DSS-induced colitis-associated CRC model, oral QJFG treatment inhibited tumor formation, reduced IL-1β, TNF-α, IL-6, and IFN-γ levels, increased circulating CD3 ^+^ T-cell abundance and the CD4 ^+^/CD8 ^+^ T-cell ratio, corrected gut microbial disturbances, and suppressed abnormal NOD2/NF-κB activation. No evident hepatic or renal toxicity was observed during long-term treatment in this model (133). A complementary study combining AOM/DSS-induced CAC mice with Ana-1 macrophage experiments showed that QJFG promoted M1-like macrophage polarization and suppressed M2-like polarization. These effects involved TLR4/MD2-mediated NF-κB, MAPK, and PI3K/Akt signaling and inhibition of IL-4R-induced STAT6 and Akt phosphorylation (134). Pathway inhibition and MD2 knockdown strengthened the functional evidence for macrophage reprogramming. These findings suggest that QJFG may improve the CRC immune microenvironment by correcting gut microbial disturbances, suppressing inflammatory NOD2/NF-κB signaling, correcting T-cell abnormalities, and reshaping TAM polarization. Notably, the reported T-cell changes were assessed in peripheral blood; whether QJFG induces comparable alterations within tumor tissues remains uncertain. Representative Chinese patent medicines and granule preparations, together with their key mechanisms in modulating the CRC immune microenvironment, are summarized in **Table 4**.

**Table 4:** Key mechanisms of Chinese patent medicines and granule preparations in modulating the CRC immune microenvironment.

Chinese patent medicine/preparation	Main immune target(s)	Key mechanism(s)	Potential application	Model system(s)	MMR/MSI	Representative dose/exposure	Evidence tier
Zhenqi Fuzheng Granule	MDSCs, macrophages, T/NK cells, gut microbiota	SCFAs–GPR109A; AKT/mTOR/HIF-1α; glycolysis suppression	Enhance PD-1 blockade	Apc^Min/+^ and AOM/DSS mice; CT26 model; FMT; RAW264.7 cells	NR	0.3–7.8 g/kg; 0.25–0.5 mg/mL	Tier 2
Huaier Granule	MHC-I, CD8 ^+^ T cells, Tregs	STAT1–MHC-I; antigen presentation; T-cell cytotoxicity	Restore immune recognition and enhance PD-1 blockade	AOM/DSS and MC38 models; CRC cells; patient-derived organoids; retrospective CRC cohort	NR	50–100 mg/mouse; 4–8 mg/mL	Tier 2
Qingjie Fuzheng Granule	TAMs, peripheral T cells, gut microbiota	NOD2/NF-κB; TLR4/MD2; IL-4R/STAT6–Akt	Prevent CAC and regulate TAM polarization	AOM/DSS mice; Ana-1/CT26 coculture; MD2 knockdown	NR	1 g/kg; 25–100 μg/mL	Tier 2

MMR/MSI status was recorded only when explicitly reported; NR denotes not reported. Dose/exposure lists representative regimens and is not directly comparable across preparations. Evidence tiers indicate the highest source directly supporting the listed immune mechanism: Tier 1, *in vitro*/in silico; Tier 2, CRC or CAC animal models; and Tier 3, immune evaluation after intervention in patients or paired human samples. Clinical use, observational outcomes, or ex vivo human models without intervention-linked human immune assessment do not qualify as Tier 3. **Tables 3**–**4** use source-based tiers because evidence for formulas and formulated products is more heterogeneous than the P1–P3 mechanistic grading used in **Tables 1**–**2**.

In summary, experimental studies suggest that Chinese herbal formulas can synergistically regulate immune cells, inflammatory signaling, tumor metabolism, stromal cells, and the gut microbiota through multi-herb compatibility and multicomponent, multitarget actions. Chinese patent medicines, with relatively fixed prescriptions and defined dosage forms, exert regulatory effects on antigen presentation, myeloid immunosuppression, and gut microbiota-associated immunometabolism. Together, these findings suggest that Chinese herbal formulas and Chinese patent medicines may regulate the CRC immune microenvironment at multiple levels. The reported effects include support of effector immunity, attenuation of immunosuppressive features, and modulation of gut microbial and metabolic abnormalities.

## 5 Conclusions and perspectives

The CRC immune microenvironment is composed of effector immune cells, immunosuppressive cells, inflammatory mediators, immune checkpoint pathways, stromal cells and the extracellular matrix, the gut microbiota, and microbial metabolites. Its dysregulation occurs across multiple stages of CRC development, from malignant transformation of colorectal adenomas to primary tumor formation and metastatic progression. Collectively, available preclinical evidence indicates that TCM-mediated regulation of the CRC immune microenvironment has gradually expanded from observations of inflammation and peripheral immune parameters to mechanistic investigations involving immune cells, immune checkpoint pathways, immunometabolism, stromal remodeling, and gut microecology. TCM-derived isolated compounds and bioactive constituents help clarify relatively specific molecular targets and immune regulatory processes, whereas single herbs, Chinese herbal formulas, and Chinese patent medicines may further modulate the interactions among tumor cells, immune cells, stromal cells, the ECM, and the gut microbiota through coordinated multicomponent effects.

**Figure 2:**
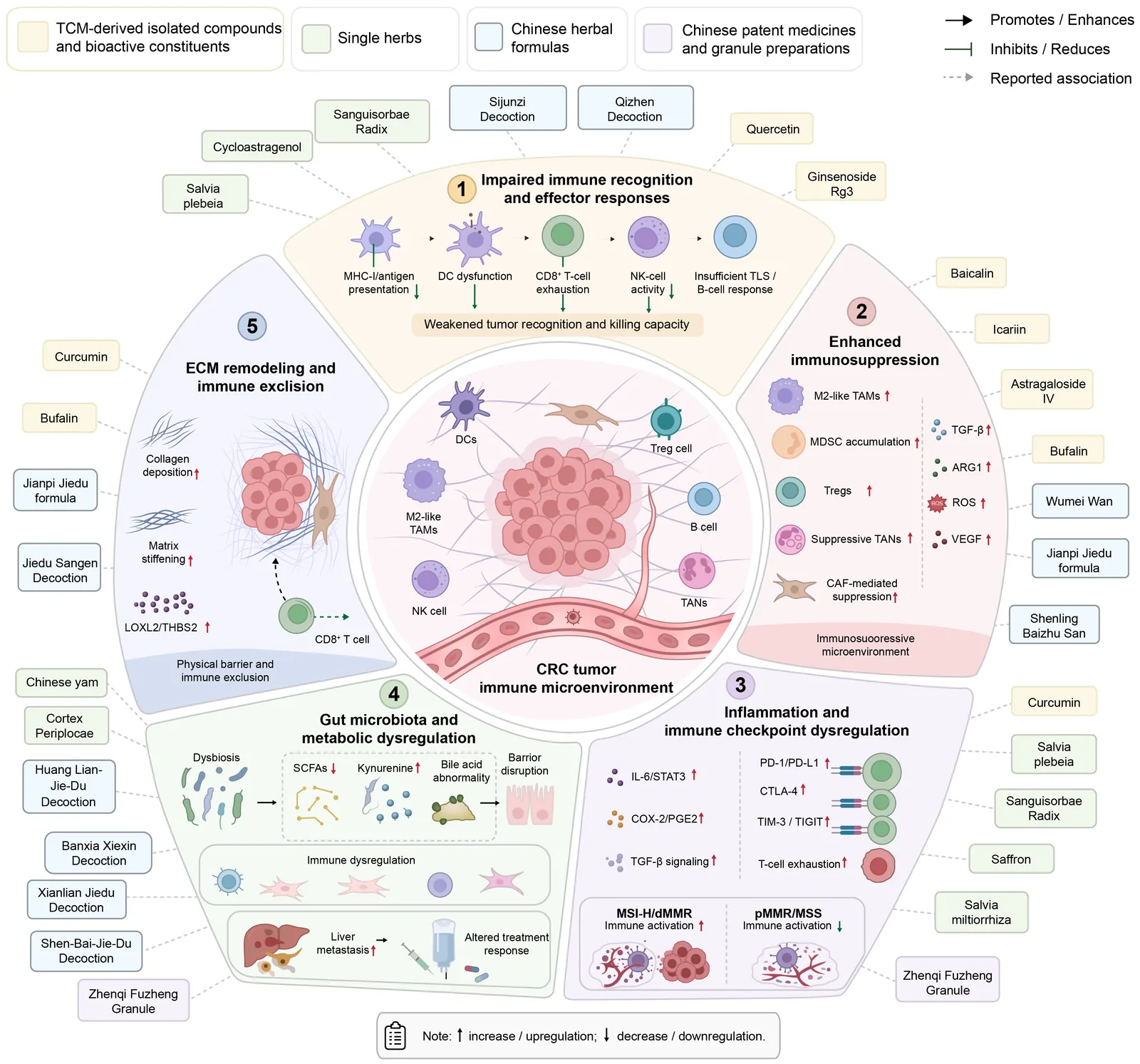
Integrated mapping of representative traditional Chinese medicine interventions onto five pathological dimensions of the CRC tumor immune microenvironment.

Based on current mechanistic evidence, TCM-mediated regulation of the CRC immune microenvironment can be summarized in five interconnected dimensions. These dimensions include restoration of immune recognition and effector immunity, attenuation of immunosuppression, intervention in stromal remodeling and immune exclusion, modulation of inflammatory signaling and immune checkpoint pathways, and reshaping of the gut microbiota and microbial metabolite profiles. Representative interventions across the four categories of TCM and their principal associations with these five dimensions are summarized in **Figure 2**. Relevant interventions can enhance MHC-I-restricted antigen presentation, induce immunogenic cell death, pyroptosis, or ferroptosis, and promote DC maturation, CD8 ^+^ T-cell responses, and NK-cell function. TCM interventions can inhibit M2-like polarization of TAMs, reduce the accumulation of MDSCs and Tregs, and attenuate TAN-associated immunosuppression. ECM remodeling is increasingly recognized as an important structural basis of immune exclusion in CRC. Accordingly, CAF activation, collagen deposition, matrix stiffening, and fibrotic barrier formation should be considered when evaluating how TCM interventions may regulate the CRC immune microenvironment. TCM interventions may also modulate inflammatory signaling and immune checkpoint pathways, including PD-1/PD-L1, alleviate T-cell exhaustion, and thereby support antitumor immune responses. Regulation of the gut microbiota, microbial metabolites, and immunometabolism constitutes the fifth dimension. Relevant processes include tryptophan and glutamine metabolism, lipid metabolism, glycolysis, and SCFA-related signaling. Changes in the gut microbiota and microbial metabolites may further influence these metabolic processes and immune remodeling in CRC. Compared with previous reviews, this review provides a complementary, pathology-oriented synthesis of five dimensions of the CRC immune microenvironment across four categories of TCM interventions. It further evaluates the evidence in relation to disease stage, molecular subtype, model relevance, and translational limitations. Representative studies of TCM interventions combined with immune checkpoint blockade in CRC are summarized in **Table 5**.

**Table 5:** Representative studies of TCM interventions combined with immune checkpoint blockade in CRC.

TCM intervention	Combined immunotherapy	Combination model(s)	Immunotherapy-related mechanism(s)	Main combination outcome
Ginsenoside Rg3-based nanoformulations	Anti-PD-L1; anti-PD-1	CT26 orthotopic CRC; MSS CRC mouse model	ROS-enhanced ICD; DC maturation; TIL recruitment	Enhanced tumor control and survival
Icariin	Anti-PD-1	MC38 subcutaneous tumors	CD8 ^+^ T-cell-derived IFN-γ-induced ferroptosis	Enhanced anti-PD-1 efficacy
Cycloastragenol	Anti-PD-1	CT26 and MC38 tumors; CRC organoids	CTSB inhibition; preservation of MHC-I antigen presentation	Enhanced CD8 ^+^ T-cell killing and tumor suppression
Curcumin	Anti-PD-1	CT26 tumors; CAF-associated xenograft model	Caspase-3/GSDME pyroptosis; CAF–TGF-β/PD-L1 suppression	Enhanced tumor suppression and survival
Honokiol–magnolol–baicalin	Anti-PD-1	MC38 orthotopic tumors	GSDME-dependent pyroptosis; CRT exposure; CD8 ^+^ T-cell recruitment	Enhanced anti-PD-1 tumor control
Sanguisorbae Radix extract	Pembrolizumab	Humanized PD-L1 MC38 tumors in humanized PD-1 mice	PD-1/PD-L1 blockade; TCR/NFAT–IL-2/PRF1 activation	Enhanced CD8 ^+^ T-cell cytotoxicity and tumor suppression
Shikonin	Anti-PD-1	CT26 subcutaneous tumors	PKM2–ROS–Hsp70; increased tumor immunogenicity	Increased DC/CD8 ^+^ T-cell infiltration and tumor suppression
Saffron actives (crocin and safranal)	PD-1 inhibitor	CT26 subcutaneous tumors	Reduced IL-17 and PD-L1; modulation of splenic T cells	Reduced tumor burden
*Salvia miltiorrhiza* aqueous extract	Anti-PD-L1	MC38 subcutaneous tumors	COX-2/PGE2 inhibition; reduced TAMs; enhanced CD8 ^+^ T-cell function	Enhanced tumor suppression
Chinese yam polysaccharide	Anti-PD-1	MC38 and CT26 subcutaneous tumors	Microbiota and deoxyguanosine regulation; reduced CD206 ^+^ M2-like macrophages; increased CD8 ^+^ T cells	Synergistic tumor suppression
Shenling Baizhu San	Tislelizumab; anti-PD-1	HCT116 tumor model; MC38-Luc liver metastasis model	Microbiota remodeling; CYP2E1–PPARα–HVA regulation; reduced Tregs and CD8 ^+^ T-cell exhaustion	Enhanced tumor control and reduced liver metastasis
Qizhen Decoction	PD-1 inhibitor	AOM/DSS CRC mice; antibiotic-treated microbiota-depleted mice	*Akkermansia*–DC–IL-12/JAK2/STAT4 signaling	Enhanced effector T-cell responses and tumor control
Zhenqi Fuzheng Granule	Anti-PD-1	AOM/DSS-induced CRC mice	SCFAs–GPR109A; suppressed AKT/mTOR/HIF-1α-mediated glycolysis; reduced MDSCs	Enhanced tumor suppression
Huaier Granule	Anti-PD-1	Patient-derived CRC organoids; MC38 tumors	STAT1–MHC-I antigen presentation; enhanced CD8 ^+^ T-cell killing	Greater tumor suppression than either monotherapy

Only CRC studies that directly evaluated TCM interventions combined with immune checkpoint blockade were included. Model systems and mechanisms refer specifically to the combination-treatment experiments.

Mechanistic studies on TCM-mediated regulation of the CRC immune microenvironment have made appreciable progress. Nevertheless, the correspondence between the complex constituents of Chinese herbal formulas and their immunological or stromal remodeling effects remains insufficiently defined, and systematic integration across different regulatory processes is still limited. Many studies still explain the pharmacological effects of TCM mainly on the basis of changes in immune cell proportions, inflammatory mediators, or selected signaling pathways. Although these findings are informative, they do not fully capture the continuous interactions among tumor cells, immune cells, stromal cells, the ECM, and the gut microbiota. TRIM29 has been implicated in CRC progression (135) and can regulate type I interferon production (136–138), PERK-mediated endoplasmic reticulum stress (139), and NLRP6/NLRP9b inflammasome activation (140) in non-CRC inflammatory or infectious models. Whether TCM remodels the CRC immune microenvironment by modulating TRIM29 expression or activity remains to be determined. Moreover, most evidence remains preclinical, independent replication and negative findings are limited, and publication bias toward positive results cannot be excluded. Future studies should place greater emphasis on: (i) integrating single-cell sequencing, spatial omics, and multi-omics approaches to define how TCM regulates distinct immune cell subsets, CAF activation, ECM remodeling, and intercellular communication, followed by rigorous experimental validation of key mechanisms; (ii) clarifying the bioactive constituents, absorbed circulating components, and dose response relationships of TCM-derived isolated compounds, single herbs, and Chinese herbal formulas, as well as the relationships between constituent combinations and their effects on immune regulation, stromal remodeling, and gut microbiota-associated metabolism; (iii) optimizing experimental models and evaluation systems by incorporating immunocompetent, orthotopic, and metastatic models, with particular attention to differences in the immune microenvironment, ECM remodeling, and immune exclusion across colorectal adenoma progression, primary tumors, metastatic lesions, and distinct molecular subtypes; and (iv) further elucidating the associations between TCM syndrome patterns and immune cell states, ECM-related features, inflammatory mediators, metabolites, and gut microbiota profiles, thereby providing a basis for stratified modulation of the CRC immune microenvironment by TCM. Finally, prospective clinical studies should evaluate clinical outcomes, immune-related biomarkers, safety, and potential interactions when TCM is combined with chemotherapy or immunotherapy.

In summary, current preclinical evidence suggests that TCM can regulate the CRC immune microenvironment at multiple levels, including immune recognition, effector immunity, immunosuppression, stromal remodeling, metabolism, and gut microecology, thereby reflecting its holistic and coordinated multilevel regulatory characteristics. Future work should integrate insights from immunology, systems biology, materials science, and artificial intelligence to strengthen mechanistic studies of TCM and support clinical study design and translational evaluation.
